# ADP‐glucose pyrophosphorylase genes, associated with kernel weight, underwent selection during wheat domestication and breeding

**DOI:** 10.1111/pbi.12735

**Published:** 2017-05-12

**Authors:** Jian Hou, Tian Li, Yamei Wang, Chenyang Hao, Hongxia Liu, Xueyong Zhang

**Affiliations:** ^1^ Institute of Crop Sciences Chinese Academy of Agricultural Sciences Beijing China

**Keywords:** *Triticum aestivum*, ADP‐glucose pyrophosphorylase, haplotype, association analysis, yield, selection

## Abstract

ADP‐glucose pyrophosphorylase, comprising two small subunits and two large subunits, is considered a key enzyme in the endosperm starch synthesis pathway in wheat (*Triticum aestivum* L.). Two genes, *TaAGP‐S1‐7A* and *TaAGP‐L‐1B*, were investigated in this study. Haplotypes of these genes were associated with thousand kernel weight (TKW) in different populations. Mean TKWs of favoured haplotypes were significantly higher than those of nonfavoured ones. Two molecular markers developed to distinguish these haplotypes could be used in molecular breeding. Frequencies of favoured haplotypes were dramatically increased in cultivars released in China after the 1940s. These favoured haplotypes were also positively selected in six major wheat production regions globally. Selection of *AGP‐S1* and *AGP‐L‐1B* in wheat mainly occurred during and after hexaploidization. Strong additive effects of the favoured haplotypes of with other genes for starch synthesis were also detected in different populations.

## Introduction

Increasing the yield of wheat (*Triticum aestivum*) will become a greater challenge in the future. Although average yields in China increased by 2.12% in the last 10 years (http://faostat3.fao.org), the annual rate of increase is gradually decreasing. Yield potential can be increased in three ways: higher spike number per unit area (SN), higher grain number per spike (GN) and higher thousand kernel weight (TKW). Crop yields at the physiological level are determined mainly by source and sink strengths (Rossi *et al*., 2015). In cereals such as wheat and barley (*Hordeum vulgare*), sink capacity has proved more important than source accumulation (Serrago *et al*., 2013); therefore, improvement in the expression of key enzymes involved in starch biosynthesis in the endosperm may be an effective way to increase yield in wheat (Bahaji *et al*., 2014; Hou *et al*., 2014).

ADP‐glucose pyrophosphorylase (AGPase) is a rate‐limiting enzyme in the synthesis of starch in plant endosperm (Dickinson and Preiss, 1969; Müller‐Röber *et al*., 1992; Tsai and Nelson, 1966), that is the catalysis of glucose‐1‐Pi to ADP‐glucose. AGPase in most plants is a tetrameric protein complex comprising two large subunits (LSUs) and two small subunits (SSUs; Copeland and Preiss, 1981; Okita *et al*., 1990). The LSUs mainly have regulatory roles and the SSUs have catalytic roles, and recent studies indicate that LSUs also contribute substrate binding (Cakir *et al*., 2015; Hwang *et al*., 2008; Kavakli *et al*., 2001). AGPases are activated by 3‐phosphoglyceric acid and inhibited by inorganic phosphate (Boehlein *et al*., 2013; Figueroa *et al*., 2013; Heldt *et al*., 1977; Neuhaus *et al*., 1989). In nonphotosynthetic cells, AGPases have different localizations in dicots and monocots (Comparot‐Moss and Denyer, 2009). AGPases are mainly active in the plastids of dicots, whereas in monocots they are active in both the cytosol and plastids, with greater activity occurring in the cytosol. The proportion of cytosolic activity in the endosperm may be as high as 85% in barley (Thorbjørnsen *et al*., 1996), 95% in maize (*Zea mays*) (Denyer *et al*., 1996) and 94% in wheat (Burton *et al*., 2002).

*AGP* mutation and overexpression are possible ways to increase yield. Heterologous expression of *Escherichia coli glgC16* in rice (*Oryza sativa*) increased seed weight by 7%–24% (Nagai *et al*., 2009; Sakulsingharoj *et al*., 2004), in maize by 13%–25% (Wang *et al*., 2007) and tuber yield in potato (*Solanum tuberosum*) by 35% (Stark *et al*., 1992). The *Shrunken2* (*Sh2*) and *Brittle2* (*Bt2*) loci in maize endosperm separately encode the large and small subunits (Hannah and Nelson, 1976). Expression of the *Sh2r6hs* allele with enhanced heat stability and reduced inorganic phosphate inhibition increased the yield of wheat by 38% (Smidansky *et al*., 2002) and of rice by 23% (Smidansky *et al*., 2003). Transgenic maize lines simultaneously overexpressing the *Bt2* and *Sh2* alleles increased starch content by 74% and seed weight by 15% compared to wild‐type (WT) lines (Li *et al*., 2011). Overexpression of LSU in wheat lines enhanced AGPase activity and increased TKW by 5.2%–9.1% compared to WT (Kang *et al*., 2013).

Most crops undergo significant genetic changes during domestication and breeding (Shi and Lai, 2015). Previous studies indicated that SSUs were more conserved than LSUs, yet both AGPase subunits are sensitive to enzyme activity that leads to amino acid changes (Georgelis *et al*., 2007) and have undergone selection in maize during domestication and breeding (Corbi *et al*., 2011). In this study, genes *TaAGP‐S1‐7A* and *TaAGP‐L‐1B* were mainly investigated. Four haplotypes of each gene were detected and were associated with TKWs when assayed in near‐isogenic lines (NILs), a mini core collection (MCC) and modern cultivars (MC). Based on their effects, these haplotypes were artificially classified into subgroups *Hap‐I* and *Hap‐II* and cleaved amplified polymorphic site (CAPS) markers were developed to identify them. There has been strong selection for the two favoured haplotypes over the past seventy years of wheat breeding in China. These favoured haplotypes also occur at high frequencies in cultivars released in other geographic regions during the last century.

## Results

### Cloning by homology and chromosome locations of *TaAGP‐S* and *TaAGP‐L*

Full‐length homologous cDNA and genomic DNA sequences of *TaAGP‐S1* (GenBank: EF405961, cytosolic), *TaAGP‐S2* (AY727927, plastidic) and *TaAGP‐L* (DQ839506, cytosolic) were cloned from Chinese Spring (CS). *TaAGP‐S1‐A* and *TaAGP‐L‐D* were located on chromosomes 7AL (scaffold53517, C‐7AL1‐0.39) and 1DL (scaffold2135, bin203), respectively, by sequence alignments to the *Triticum urartu* and *Aegilops tauschii* genome sequences. *TaAGP‐S1*,* TaAGP‐S2* and *TaAGP‐L* were located on homoeologous chromosome groups 7, 5 and 1, respectively, and verified by PCR amplification of DNA from CS and its nulli‐tetrasomic lines with genome‐specific primers (Table S1).

Sequence lengths of coding regions of *TaAGP‐S1‐7A*,* ‐7B* and *‐7D* were 7531, 6245 and 6072 bp, respectively; each had nine exons and eight introns. *TaAGP‐S1‐7A* has an alternative first exon in the first intron that encodes plastid SSU in leaves (Comparot‐Moss and Denyer, 2009), and also has a 1.1‐kb insertion in the first intron compared to *‐7B* and *‐7D*. This inserted region had a GC content above 70% and was also present with high homology in *AGP‐S1* in *T. urartu*.

Sequence lengths of the coding regions of *TaAGP‐S2‐5A*,* ‐5B* and *‐5D* were 3732, 3774 and 3771 bp, respectively; each had nine exons and eight introns. Sequence lengths of coding regions of *TaAGP‐L‐1A*,* ‐1B* and *‐1D* were 3332, 3351 and 3340 bp, respectively; each had 15 exons and 14 introns. Although the three genes within each homoeologous group had homologies above 92%, the small and large subunit genes have very low homology at both the cDNA (52%–54%) and DNA (40%) levels (Figure S1).

### Genetic haplotypes and their effects on TKW in a MCC and NILs

**Figure 1:**
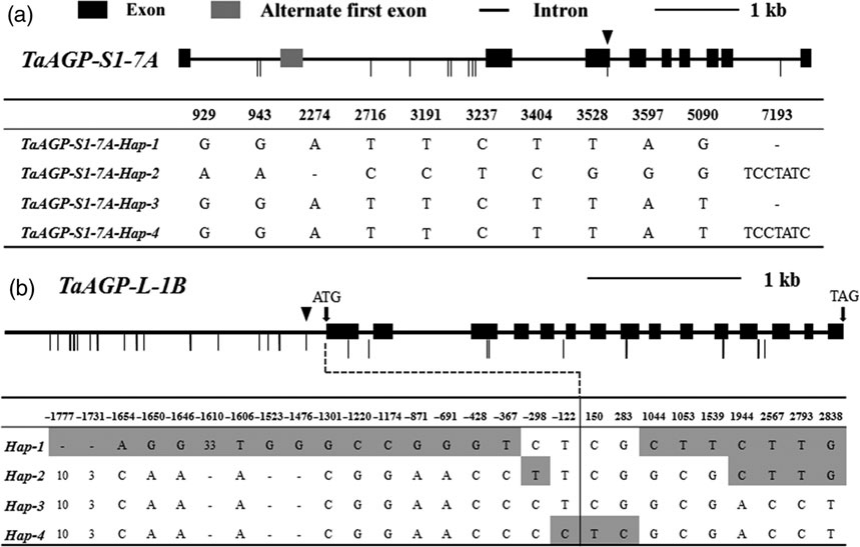
Haplotypes of *TaAGP‐S1‐7A* and *TaAGP‐L‐1B*. (a) Coding regions of *TaAGP‐S1‐7A*. ▼SNP at position 5090. (b) Coding and 2‐kb upstream regions, and polymorphic sites of *TaAGP‐L‐1B*. ▼SNP at position ‐122. Numbers indicate deletion size (bp). Vertical thin lines indicate polymorphic site differences between haplotypes.

We sequenced *TaAGP‐S1*,* TaAGP‐S2* and *TaAGP‐L* in 245 accessions from the MCC, and polymorphic sites were detected only in *TaAGP‐S1‐7A* and *TaAGP‐L‐1B* (Figure 1).

**Figure 2:**
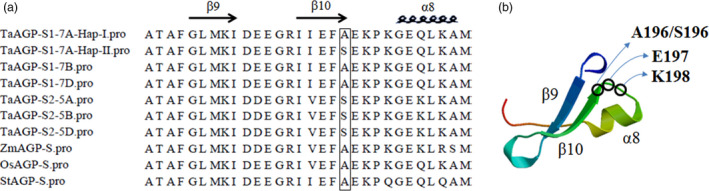
Key polymorphic sites on protein sequences of TaAGP‐S1‐7A‐Hap‐I and ‐Hap‐II. (a) Partial amino acid (aa) sequence alignments of TaAGP‐S1, TaAGP‐S2, ZmAGP‐S (maize), OsAGP‐S (rice) and StAGP‐S (potato). Key site differences between TaAGP‐S1‐7A‐Hap‐I and ‐Hap‐II are in the rectangle. (b) Secondary protein structure of StAGP‐S (30 aa) predicted by the SWISS‐MODEL (Arnold *et al*., 2006; Guex and Peitsch, 1997; Schwede *et al*., 2003).

A SNP at position 5090 in the third exon of *TaAGP‐S1‐7A* (Figure 1a) led to an amino acid change (GCA→TCA, Ala→Ser). Dendrogram analysis showed that this site was Ala in most species and the amino acid sequences around it were highly homologous (Sarma *et al*., 2014). In potato, this site was A196 and next to E197 and K198 in AGP‐L (Jin *et al*., 2005). E197 and K198 are essential for binding substrate ADP‐glucose. Therefore, the change from Ala to Ser might result in lower enzyme activity associated with an allosteric change (Figure 2). Interestingly, this site was also Ser in plastid TaAGP‐S2‐5A, ‐5B and ‐5D. In a previous study, the activities of cytosolic SSUs in wheat endosperm reached 94%, and activities of plastid SSUs accounted for only 6% of the total (Burton *et al*., 2002). Thus, this SNP might cause reduced enzyme activity.

Another eight SNPs and two insertions/deletions (InDels) were identified among the 245 sequenced accessions, forming four haplotypes, named *‐Hap‐1*,* ‐Hap‐2*,* ‐Hap‐3* and *‐Hap‐4* (Figure 1a). The numbers of accessions in the *‐Hap‐2* and *‐Hap‐4* groups were quite small, at four and four in landraces compared to nine and zero in modern cultivars. Only the SNP (G/T) at position 5090 in the third exon caused an amino acid difference between *‐Hap‐1/2* and *‐Hap‐3/4*; other polymorphic sites were all in introns. Significant differences in TKW were detected between *‐Hap‐1* and *‐Hap‐3* in both landraces and modern cultivars of the MCC (Table 1; Figure S2a). There were no significant differences in SN, GN and TKW between *‐Hap‐1* and *‐Hap‐2* in 2014 plot tests (Table S2; Figure S3a). The relative expression level of *‐Hap‐1* was not significantly different from that of *‐Hap‐2* at 15 days postanthesis (DPA) (Table S3; Figure S3b), suggesting that *‐Hap‐1* and *‐Hap‐2* had similar effects on TKW.

**Table 1:** Mean TKWs of *TaAGP‐S1‐7A* and *TaAGP‐L‐1B* haplotypes and their combinations in the MCC

	Landraces in MCC				Modern cultivars in MCC			
	Number of accessions	2002	2005	2006	Number of accessions	2002	2005	2006
*TaAGP‐S1‐7A*								
*Hap‐1*	28	34.53 ± 1.20a	34.17 ± 1.54a	35.66 ± 1.41a	60	40.55 ± 0.58a(A)	40.23 ± 0.70a(A)	42.52 ± 0.72a(A)
*Hap‐2*	4	31.56 ± 1.06ab	28.40 ± 0.97ab	32.51 ± 1.41ab	9	37.81 ± 1.76ab(AB)	35.28 ± 2.70ab(AB)	38.88 ± 1.92ab(AB)
*Hap‐3*	120	31.58 ± 0.38b	29.94 ± 0.54b	32.41 ± 0.44b	19	35.71 ± 1.09b(B)	34.24 ± 1.27b(B)	36.33 ± 1.27b(B)
*Hap‐4*	4	32.83 ± 0.77ab	30.86 ± 0.98ab	34.88 ± 0.29ab				
*Hap‐I*	32	34.16 ± 1.07a	33.35 ± 1.38a(A)	35.27 ± 1.26a	69	40.19 ± 0.56a(A)	39.70 ± 0.71a(A)	42.05 ± 0.68a(A)
*Hap‐II*	124	31.62 ± 0.36b	29.97 ± 0.53b(B)	32.49 ± 0.43b	19	35.71 ± 1.09b(B)	34.24 ± 1.27b(B)	36.33 ± 1.27b(B)
*TaAGP‐L‐1B*								
*Hap‐1*	23	34.16 ± 0.98a	34.49 ± 1.16a(AB)	36.09 ± 1.16a(A)	22	41.19 ± 1.08a	39.93 ± 1.28a	43.07 ± 1.06a
*Hap‐2*	9	35.77 ± 2.50a	37.86 ± 2.92a(B)	36.30 ± 2.79ab(AB)	22	39.53 ± 0.86ab	38.80 ± 1.10a	41.22 ± 1.00ab
*Hap‐3*	30	32.09 ± 0.56ab	30.18 ± 0.83b(AC)	33.01 ± 0.67ab(AB)	30	38.50 ± 0.90ab	38.79 ± 1.19a	40.22 ± 1.23ab
*Hap‐4*	65	30.98 ± 0.49b	28.82 ± 0.59b(C)	31.58 ± 0.57b(B)	13	37.23 ± 1.56b	36.00 ± 2.09a	37.88 ± 1.93b
*Hap‐I*	62	32.97 ± 0.51a(A)	31.90 ± 0.73a(A)	34.10 ± 0.60a(A)	74	39.60 ± 0.56a	39.14 ± 0.68a	41.37 ± 0.67a
*Hap‐II*	65	30.98 ± 0.49b(B)	28.82 ± 0.59b(B)	31.58 ± 0.57b(B)	13	37.23 ± 1.56a	36.00 ± 2.09a	37.88 ± 1.93b
SI+LI	29	34.48 ± 1.14a(A)	34.07 ± 1.47a(A)	35.64 ± 1.33a(A)	61	40.32 ± 0.59a(A)	39.94 ± 0.73a(A)	42.25 ± 0.71a(A)
SI+LII	3	31.05 ± 2.48a(A)	27.30 ± 1.76a(A)	31.62 ± 3.87a(A)	8	39.20 ± 1.85ab(AB)	37.73 ± 2.79ab(AB)	40.52 ± 2.46ab(AB)
SII+LI	62	32.26 ± 0.52a(A)	30.94 ± 0.82a(A)	33.39 ± 0.63a(A)	13	36.22 ± 1.16b(B)	34.56 ± 1.23b(B)	37.24 ± 1.42b(B)
SII+LII	62	30.98 ± 0.50b(B)	28.90 ± 0.62b(B)	31.58 ± 0.58b(B)	6	34.60 ± 2.45b(B)	33.60 ± 3.14b(AB)	34.36 ± 2.63b(B)

SI, SII: *TaAGP‐S1‐7A‐Hap‐I* (*‐Hap‐1* and *‐2*), *‐Hap‐II* (*‐Hap‐3* and *‐4*). LI, LII: *TaAGP‐L‐1B‐Hap‐I* (*‐Hap‐1*,* ‐2* and *‐3*), *‐Hap‐II* (*‐Hap‐4*).

Different capital and small letters indicate significant differences between haplotypes at *P *< 0.01 and *P *< 0.05, respectively.

Nine SNPs in the coding region, and 13 SNPs and five InDels in the 5′ UTR and promoter regions of *TaAGP‐L‐1B* formed four haplotypes, *‐Hap‐1*,* ‐Hap‐2*,* ‐Hap‐3* and *‐Hap‐4* (Figure 1b). No amino acid change was detected within any of the four haplotypes. Significant differences in TKW were detected between *‐Hap‐1* and *‐Hap‐4* in landraces and modern cultivars (Table 1; Figure S2b), whereas no significant differences were detected between *‐Hap‐1*,* ‐Hap‐2* and *‐Hap‐3*. SN, GN, and TKW data for *‐Hap‐1* and *‐Hap‐3* in 2014 plot tests were not significantly different (Table S2; Figure S4a). The relative expression level of *‐Hap‐1* was not significantly different from that of *‐Hap‐3* at 20 DPA (Table S3; Figure S4b). TKW of *‐Hap‐1* was not significantly different from *‐Hap‐2*, but was significantly higher than *‐Hap‐4* by 6.0 and 6.8 g in 2011 and 2012 plot tests (Table S2; Figure S4c, d).

In the case of *TaAGP‐S1‐7A*,* ‐Hap‐1* and *‐Hap‐2* were combined as *‐Hap‐I*, and *‐Hap‐3* and *‐Hap‐4* were combined as *‐Hap‐II* due to their similar effects. *TaAGP‐L‐1B‐Hap‐1*,* ‐Hap‐2* and *‐Hap‐3* were classified as *‐Hap‐I*, and *‐Hap‐4* became *‐Hap‐II*. The TKW of *‐Hap‐I* was significantly higher than that of *‐Hap‐II* in landraces and modern cultivars (Table 1; Figure S2c, d). The frequency of *TaAGP‐S1‐7A‐Hap‐I* was 20.5% in landraces compared to 78.4% in modern cultivars, and the frequency of *TaAGP‐L‐1B‐Hap‐I* was 48.8% in landraces compared to 85.1% in modern cultivars.

### The distinct haplotype effect of *TaAGP‐L‐1B* was caused by variation in one SNP in the promoter region

There were three SNPs between *‐Hap‐I* and *‐Hap‐II*, at positions −122 (T–C), 150 (C–T) and 283 (G–C) in *TaAGP‐L‐1B*. The SNP at position 150 involved a synonymous mutation (AAC‐AAT) in the first exon, position 283 was in the first intron, and position −122 was located in an E2F‐DP‐binding motif (WTTSSCSS). Rose *et al*. (2015) reported that 47 wheat cultivars genotyped at *TaAGP‐L‐1B* consisted of five haplotypes, and H1 to H4 in their study could be regarded as ‐*Hap‐I* in the present research. The SNP at position 182 (−122 in this study) was not mentioned because this SNP was present only at H5 in their study, and only in one cultivar (Chinese Spring).

**Figure 3:**
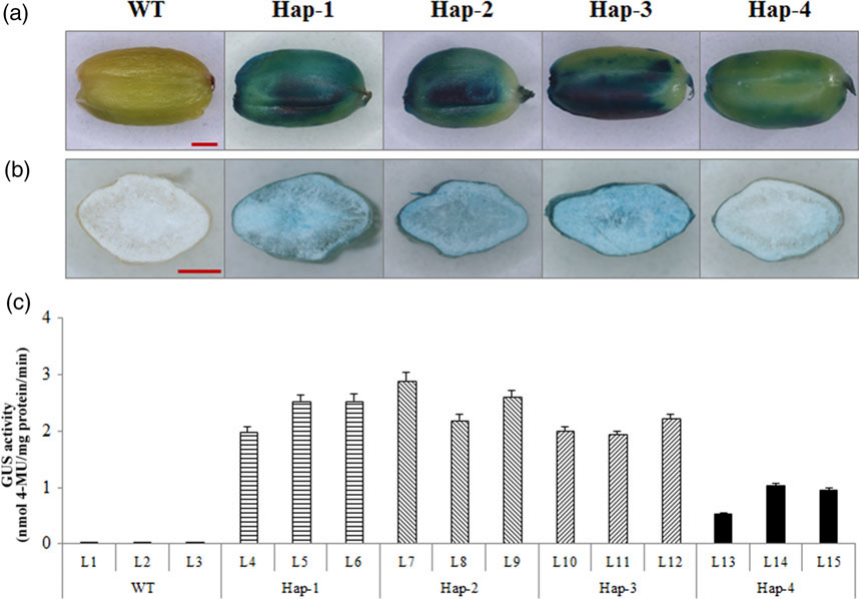
GUS staining and expression driven by the promoters of *TaAGP‐L‐1B‐Hap‐1*,* ‐Hap‐2*,* ‐Hap‐3* and *‐Hap‐4* in developing seeds at 21 DPA. (a) GUS staining of whole seeds in wild type (WT) and four haplotypes. (b) GUS staining of half‐seeds in WT and four haplotypes. (c) *GUS* activities in seeds of WT and four haplotypes.

To detect whether the SNP at position −122 caused a difference, rice seeds transformed with *‐Hap‐I* and *‐Hap‐II* promoter‐*GUS* were employed to measure promoter‐driven activity. *Gus* expression was mainly located in the aleurone layer at 21 DPA (Figure 3a, b), as also reported by Thorneycroft *et al*. (2003). *Gus* activity in developing seeds transformed with ‐*Hap‐I* was significantly higher than in those transformed with ‐*Hap‐II* (Table S4; Figure 3c). This suggested that the SNP difference at position −122 caused the phenotypic variation.

### Haplotype effects associated with TKW and their selection in the MC

**Figure 4:**
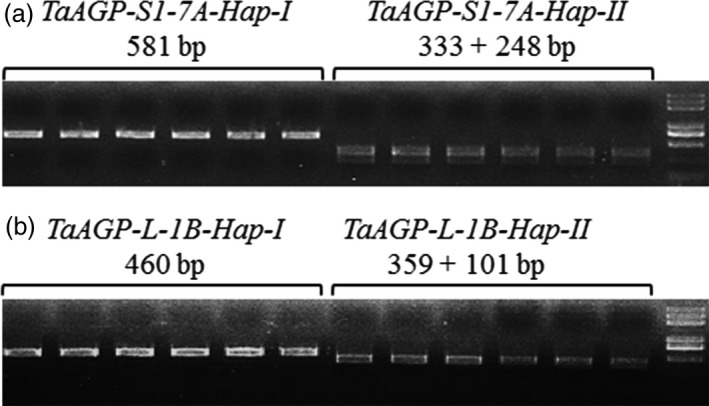
CAPS markers discriminating *TaAGP‐S1‐7A* and *TaAGP‐L‐1B* haplotypes. PCR products were restrictively digested by *Dde*I (a) and *BSr* *D*I (b).

Two cleaved amplified polymorphic site (CAPS) markers based on restriction enzyme digestion were developed to distinguish each haplotype. Position 5090 of *TaAGP‐S1‐7A‐Hap‐II* (T) fell within the restriction site CTNAG that was cleaved by restriction endonuclease *Dde*I (Figure 4a) although about 3.0 kb of sequence around the SNP was highly conserved. A 3.3‐kb genome‐specific fragment was amplified by primers S1‐7A‐M1F and S1‐7A‐M1R in the first step, and a 581‐bp fragment was amplified by primers S1‐7A‐M2F and S1‐7A‐M2R using the PCR products as template following a 25× dilution. The 581‐bp *TaAGP‐S1‐7A‐Hap‐II* fragment was digested into 333‐ and 248‐bp subcomponents by *Dde*I, whereas the *‐Hap‐I* fragment was not cleaved. Position 150 of *TaAGP‐L‐1B‐Hap‐II* (T) fell within the restriction site GCAATGNN that was cleaved by *BSrD*I (Figure 4b). The 460‐bp genome‐specific fragment amplified by primers L‐1B‐MF and L‐1B‐MR was cleaved into 359‐ and 101‐bp subfragments by *BSrD*I, whereas *‐Hap‐I* was not cleaved.

**Figure 5:**
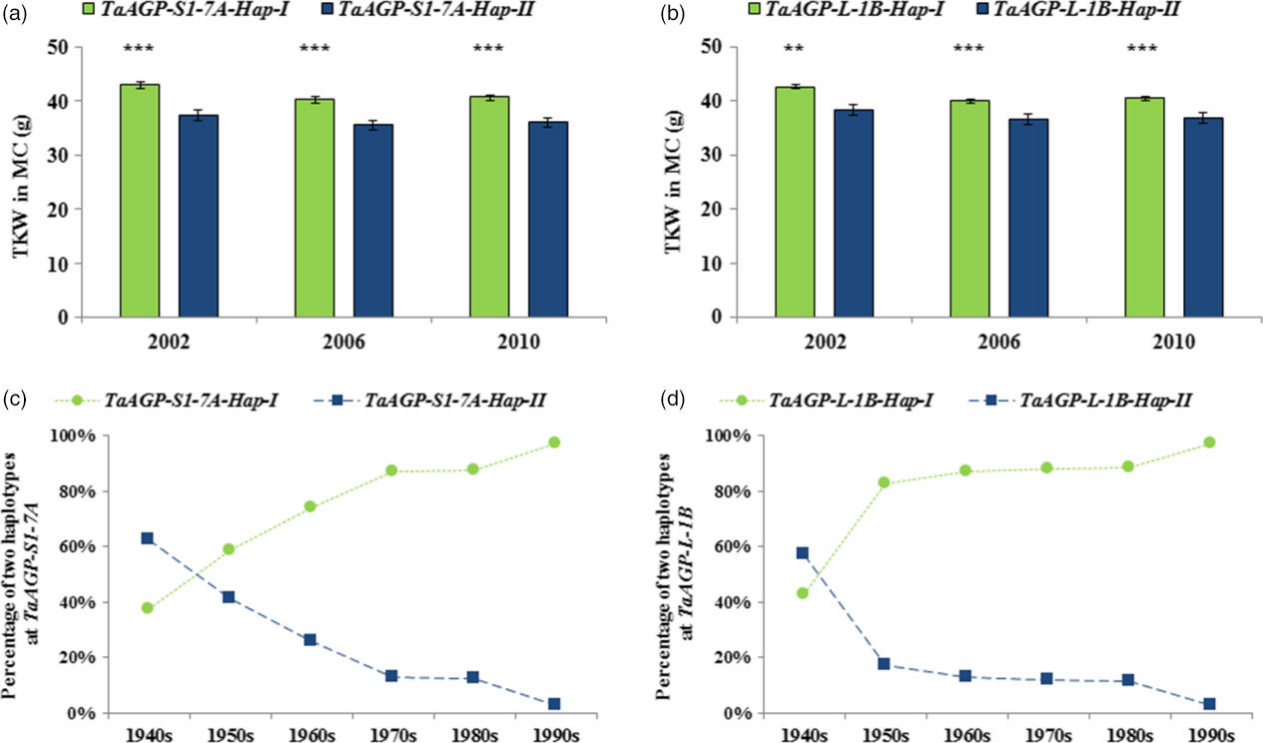
Mean TKWs of two genes in the MC. (a) TKWs of *TaAGP‐S1‐7A‐Hap‐I* and *‐Hap‐II*. (b) TKWs of *TaAGP‐L‐1B‐Hap‐I* and *‐Hap‐II*. (c) *TaAGP‐S1‐7A‐Hap‐I* and *‐Hap‐II* frequencies in cultivars released in China from the 1940s to 2010. (d) Frequencies of *TaAGP‐L‐1B‐Hap‐I* and *‐Hap‐II*. **P < 0.01, ***P < 0.001.

When 348 MC accessions were assayed by the two CAPS markers, the mean TKW of *TaAGP‐S1‐7A‐Hap‐I* was significantly higher than that of *‐Hap‐II* by 5.7 g in 2002, 4.9 g in 2006 and 4.7 g in 2010 (Table 2; Figure 5a). The TKW of *TaAGP‐L‐1B‐Hap‐I* was significantly higher than *‐Hap‐II* by 4.2 g in 2002, 3.5 g in 2006 and by 3.7 g in 2010 (Table 2; Figure 5b). Each ‐*Hap‐I* was defined as a favoured haplotype relative to the corresponding ‐*Hap‐II*. The proportion of favoured haplotypes gradually rose during six decades of breeding, reaching 97% in the 1990s (Figure 5c, d).

**Table 2:** Mean TKWs of *TaAGP‐S1‐7A* and *TaAGP‐L‐1B* haplotypes and their combinations in the MC

	Number of accessions	2002	2006	2010
*TaAGP‐S1‐7A*				
*Hap‐1*	248	43.16 ± 0.40a(A)	40.69 ± 0.39a(A)	40.82 ± 0.39a(A)
*Hap‐2*	23	42.05 ± 1.15ab(AB)	37.39 ± 1.52b(AB)	39.71 ± 1.04ab(AB)
*Hap‐3*	58	37.32 ± 0.89b(B)	35.50 ± 0.96b(B)	36.01 ± 0.79b(B)
*Hap‐I*	271	43.07 ± 0.38a(A)	40.41 ± 0.38a(A)	40.72 ± 0.36a(A)
*Hap‐II*	58	37.32 ± 0.89b(B)	35.50 ± 0.96b(B)	36.01 ± 0.79b(B)
*TaAGP‐L‐1B*				
*Hap‐1*	130	40.74 ± 0.52a(A)	43.08 ± 0.53a(A)	40.84 ± 0.56a(A)
*Hap‐2*	79	39.91 ± 0.86a(A)	42.53 ± 0.73a(A)	40.77 ± 0.66a(A)
*Hap‐3*	92	39.04 ± 0.75ab(AB)	41.77 ± 0.78a(AB)	39.62 ± 0.69a(AB)
*Hap‐4*	44	36.55 ± 0.95b(B)	38.33 ± 1.07b(B)	36.78 ± 0.94b(B)
*Hap‐I*	301	42.53 ± 0.38a(A)	40.01 ± 0.39a(A)	40.44 ± 0.36a(A)
*Hap‐II*	44	38.33 ± 1.07b(B)	36.55 ± 0.95b(B)	36.78 ± 0.94b(B)
SI+LI	245	43.41 ± 0.39a(A)	40.67 ± 0.41a(A)	41.04 ± 0.39a(A)
SI+LII	24	39.73 ± 1.31ab(AB)	37.70 ± 0.99b(AB)	38.20 ± 1.07ab(AB)
SII+LI	41	37.82 ± 1.02b(B)	35.76 ± 1.13b(B)	36.34 ± 0.96b(B)
SII+LII	16	36.36 ± 1.86b(B)	34.71 ± 2.01b(B)	35.44 ± 1.53b(B)

SI, SII: *TaAGP‐S1‐7A‐Hap‐I* (*‐Hap‐1* and *‐2*), *‐Hap‐II* (*‐Hap‐3* and *‐4*). LI, LII: *TaAGP‐L‐1B‐Hap‐I* (*‐Hap‐1*,* ‐2* and *‐3*), *‐Hap‐II* (*‐Hap‐4*).

Different capital and small letters indicate significant differences between haplotypes at *P *< 0.01 and *P *< 0.05, respectively.

### Haplotype combinations and selection within the MCC and MC

To determine whether there were additive effects of favoured haplotypes at the two loci, we combined haplotypes and tested their effects on TKW in the two collections. *TaAGP‐S1‐7A‐Hap‐I* and *‐Hap‐II* were assigned as S‐I and S‐II, and *TaAGP‐L‐1B‐Hap‐I* and *‐Hap‐II* were named as L‐I and L‐II, respectively. The mean TKW of the S‐I + L‐I cohort was significantly higher than that of S‐II + L‐II in both populations, and the proportion of accessions with the favoured combination also gradually rose over time (Table 1; Figure S5). TKW differences among the four haplotype combinations clearly indicated the existence of a genetically additive effect of the favoured haplotypes.

### Geographic distributions of haplotypes and additive effects with other important starch synthesis genes in world cultivar populations

Frequencies of favoured haplotypes at *TaAGP‐S1‐7A* and *TaAGP‐L‐1B* were assessed by CAPS markers in 384 European, 447 North American, 53 CIMMYT, 82 Russian and 51 Australian cultivars (Figure S6a,b). These six regions account for about 60% of global wheat production (http://faostat3.fao.org). Favoured haplotypes at *TaAGP‐S1‐7A* and *TaAGP‐L‐1B* reached very high frequencies (from 82% to 100%) in all five groups, indicating they had undergone overall positive selection in wheat breeding.

We previously analysed genes encoding sucrose synthase I and II in the starch synthesis pathway, and determined their haplotypes and global distributions in cultivars released during a century of wheat breeding (Hou *et al*., 2014). Haplotype effects on TKW between favoured and nonfavoured haplotypes at six loci (*TaAGP‐S1‐7A*,* TaSus1‐7B*,* TaAGP‐L‐1B*,* TaSus2‐2A*,* TaSus1‐7A* and *TaSus2‐2B*) were compared in the MC (Figure S6c). The largest difference was found at *TaAGP‐S1‐7A*. The frequencies of favoured haplotypes reached very high levels at all loci in cultivars released in Europe, North America and China (Figure S6d).

**Figure 6:**
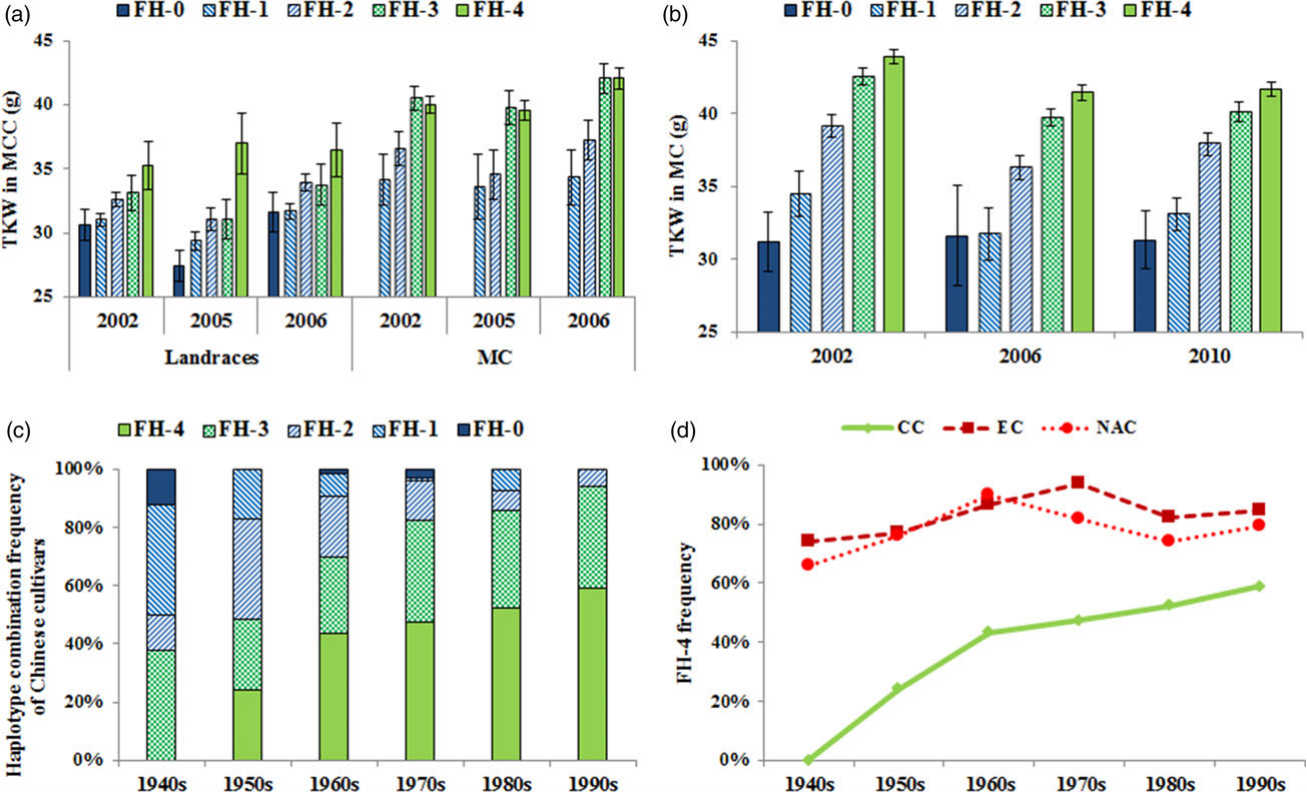
TKW effects and frequency changes of cultivars with different numbers of favoured haplotypes (FH) at *TaAGP‐S1‐7A*,* TaSus1‐7B*,* TaAGP‐L‐1B* and *TaSus2‐2A* in different populations. (a) TKW differences in cultivars with different FH in the MCC. (b) TKW differences in the MC accessions. (c) Frequency changes among accessions with different FH in the MC over six decades. (d) Frequency changes among accessions with four favoured haplotypes in Chinese cultivars (CC), European cultivars (EC) and North American cultivars (NAC).

As expected, cultivars with more favoured haplotypes had higher mean TKWs in the MC and MCC (Table 3; Figure 6a, b). The frequencies of cultivars with four favoured haplotypes at *TaAGP‐S1‐7A*,* TaSus1‐7B*,* TaAGP‐L‐1B* and *TaSus2‐2A* increased during six decades of Chinese, European and North American wheat improvement (Figure 6c, d), with Chinese cultivars rising fastest.

**Table 3:** Frequency of favoured haplotypes (FH) and mean TKWs in the MCC and MC

Population	Number of FH	Number of accessions	2002	2005	2006	Population	Number of FH	Number of accessions	2002	2006	2010
Landraces in the MCC	0	9	30.65 ± 1.22ab	27.43 ± 1.23a(A)	31.61 ± 1.50ab	MC	0	6	31.18 ± 3.45a(AB)	31.60 ± 2.03a(A)	31.32 ± 1.99a(A)
	1	65	31.06 ± 0.50a	29.35 ± 0.67a(A)	31.68 ± 0.58a		1	21	34.45 ± 1.78a(B)	31.74 ± 1.55a(A)	33.08 ± 1.14a(AB)
	2	52	32.62 ± 0.55ab	31.09 ± 0.86a(AB)	33.90 ± 0.67ab		2	46	39.15 ± 0.80a(BC)	36.30 ± 0.75b(AB)	37.93 ± 0.78ab(AB)
	3	17	33.09 ± 1.35ab	31.03 ± 1.54ab(AB)	33.72 ± 1.61ab		3	114	42.54 ± 0.57b(AC)	39.75 ± 0.62c(BC)	40.11 ± 0.64bc(C)
	4	14	35.26 ± 1.86b	36.97 ± 2.33b(B)	36.45 ± 2.10b		4	157	43.92 ± 0.51b(A)	41.45 ± 0.50c(C)	41.67 ± 0.45c(C)
Modern cultivars in the MCC	1	8	34.12 ± 1.98a(A)	33.62 ± 2.57a	34.33 ± 2.17a(A)						
	2	10	36.55 ± 1.30ab(AB)	34.54 ± 1.96a	37.26 ± 1.56ab(AB)						
	3	24	40.50 ± 0.99b(B)	39.79 ± 1.30a	42.07 ± 1.15b(B)						
	4	46	40.03 ± 0.67b(B)	39.57 ± 0.82a	42.06 ± 0.83b(B)						

Different capital and small letters indicate significant differences between haplotypes at *P *< 0.01 and *P *< 0.05, respectively.

### Relationship between ancestral species and hexaploid accessions

Fifteen diploid and 36 tetraploid wheat accessions were genotyped at *AGP‐S1*. Distinct differentiation occurred between *T. urartu* and *T. boeoticum* (*T. monococcum*). Polyploid wheats were closer to *T. urartu*, supporting the currently held opinion that *T. urartu* is the A genome donor to polyploid wheats (Feldman and Levy, 2015). When neglecting polymorphic sites that were less than 10% in frequency, most tetraploid accessions clustered into three haplotype groups (Figure S7). Hap‐1 and Hap‐2 had the same polymorphic sites as *TaAGP‐S1‐7A‐Hap‐1* and *‐Hap‐2*, but Hap‐3 was detected only in tetraploid wheat, indicating that it was lost during hexaploidization of common wheat.

**Figure 7:**
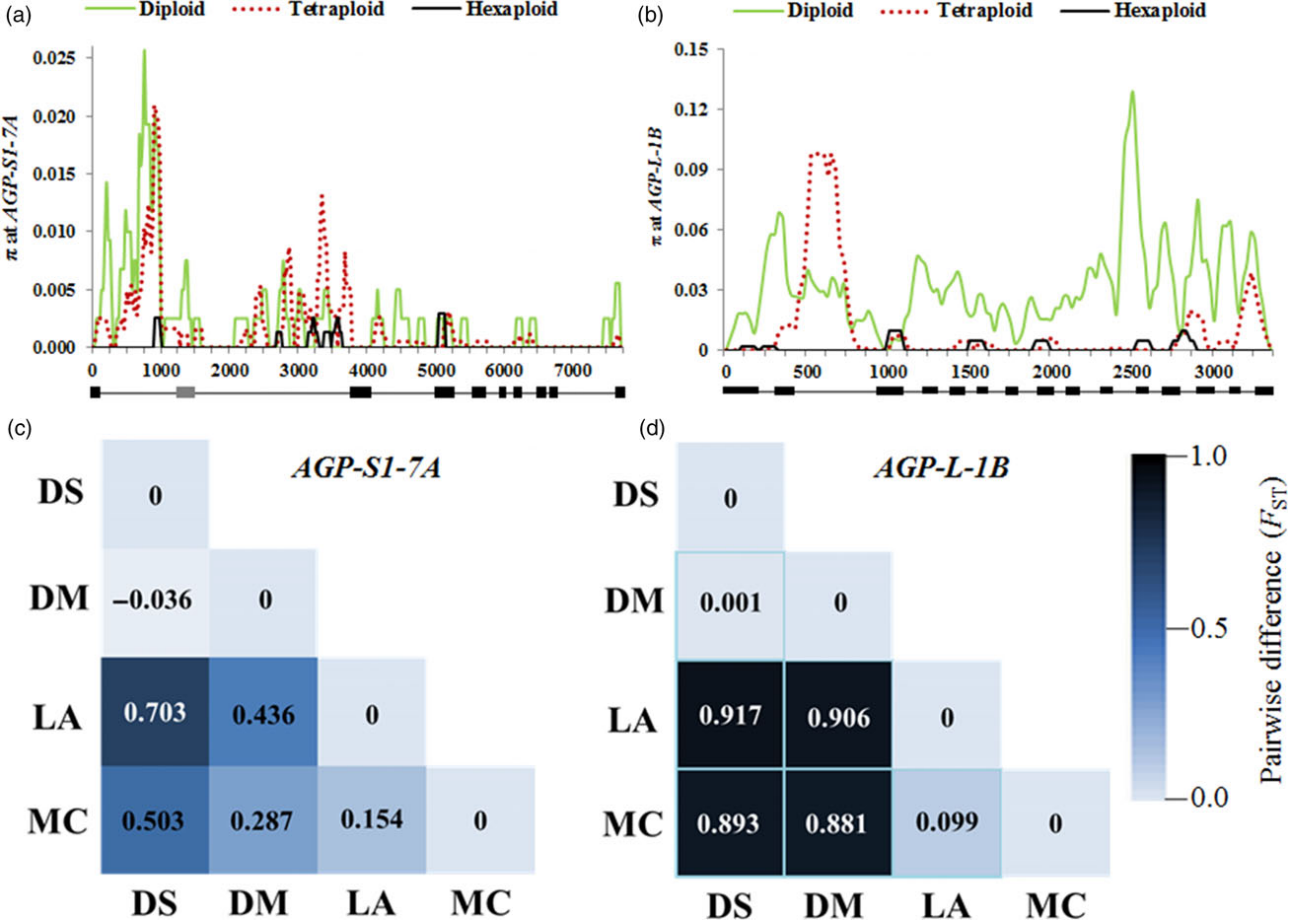
Nucleotide polymorphisms (π) and genetic differentiation ( *F*_ST_ ) between pairs of populations at *AGP‐S1‐7A* and *AGP‐L‐1B*. (a) π values in diploid, tetraploid and hexaploid accessions at *AGP‐S1‐7A*; the line under the horizontal axis represents the coding region. (b) π values in diploid, tetraploid and hexaploid accessions at *AGP‐L‐1B*. (c) *F*_ST_ in DS, DM, LA and MC at *AGP‐S1‐7A*, and blue colour gradient represents changes *F*_ST_ values from dark (1.0) to light (0.0). (b) *F*_ST_ in DS, DM, LA and MC at *AGP‐L‐1B*. DS,* Triticum dicoccoides*; DM, other tetraploid accessions used in the study; LA, landraces in the MCC; MC, Chinese modern cultivars in the MCC.

Nucleotide diversity (π) dramatically declined with polyploidization (Figure 7a). The π_D_/π_T_ was only 1.43, but π_T_/π_H_ was 9.11 (Table 4). Pairwise differences between populations (*F*_ST_) showed that the *F*_ST_ between cultivated and wild tetraploid wheats was only −0.036, but this value reached 0.436 between hexaploid landraces in the MCC and cultivated tetraploid wheat. This value was 0.154 when modern cultivars and landraces in the MCC were compared (Figure 7c). These results indicated that selection on *AGP‐S1‐7A* mainly occurred during and after hexaploidization.

**Table 4:** Diversity and Tajima tests at the *AGP‐S1‐7A* and *AGP‐L‐1B* loci

	π	θ	Tajima's *D*	*P*
*AGP‐S1‐7A*				
Diploid accessions	0.00234	0.00344	−1.72416	<0.05
Tetraploid accessions	0.00164	0.00338	−1.90879	<0.05
Hexaploid accessions	0.00018	0.00019	−0.11657	>0.10
*AGP‐L‐1B*				
*Aegilops speltoides*	0.03122	0.02525	0.96839	>0.10
Tetraploid accessions	0.00817	0.00643	1.01117	>0.10
Hexaploid accessions	0.00113	0.00042	3.61558	<0.001

Diversity at *AGP‐L‐1B* was higher than that at *AGP‐S1‐7A* (Table 4). It also declined dramatically during polyploidization (π_D_/π_T_ = 3.82, π_T_/π_H_ = 7.23; Figure 7b). *F*_ST_ between the cultivated and wild tetraploid wheat was only 0.001, yet this value reached 0.906 between hexaploid landraces and cultivated tetraploid wheat. However, this value was only 0.099 between modern cultivars and common wheat landraces (Figure 7d). These data indicated that strong selection on *AGP‐L‐1B* had occurred during hexaploidization, domestication and breeding of common wheat. Tajima's *D* index also supports this opinion (*P *< 0.001).

## Discussion

### Haplotype differences at *TaAGP‐S1‐7A* and *TaAGP‐L‐1B* are associated with variation in TKW

AGPases synthesize the starch substrate ADP‐glucose. AGPases are ancestral plastidial enzymes expressed in photosynthetic tissues, but are highly expressed in the developing endosperm of grasses due to duplication of the AGPase multigene family (Comparot‐Moss and Denyer, 2009). In this study, we focused on genes *TaAGP‐S1‐7A* and *TaAGP‐L‐1B* expressed in developing wheat endosperm. Four haplotypes of each gene were detected in modern cultivars. Some haplotypes at each locus showed similar expression patterns and no variation in TKW in the MCC and NILs (Figures S2, S3, S4). These haplotypes were pooled into single subgroups based on their similar phenotypic effects, and polymorphic sites were reduced to one at *TaAGP‐S1‐7A* and three at *TaAGP‐L‐1B*. These sites might be causative SNPs affecting TKW.

### The SNP at position −122 of *TaAGP‐L‐1B* is associated with variation at the transcript level

The SNP at position −122 of *TaAGP‐L‐1B‐Hap‐II* is within an E2F‐DP‐binding motif (WTTSSCSS) and therefore may change *TaAGP‐L‐1B* into an E2F target gene. This motif is not present in the 1‐kb sequence upstream of ‐*Hap‐I*. E2F transcription factors can be inhibitors or activators of E2F target genes in *Arabidopsis thaliana* (Mariconti *et al*., 2002; Vandepoele *et al*., 2002). Proteins encoded by plant E2F target genes are involved in cell cycle regulation, DNA replication and repair, chromatin dynamics and many other functions (Vandepoele *et al*., 2005). In this study, seeds transformed with *TaAGP‐L‐1B‐Hap‐II* promoter‐*GUS* had lower *GUS* expression (Figure 3), suggesting this nonfavoured haplotype generated a lower transcript level due to the E2F motif.

### The *TaAGP‐S1‐7A* and *TaAGP‐L‐1B* loci underwent strong selection in both domestication and breeding of common wheat

The extreme difference between the π_D_/π_T_ and π_T_/π_H_ ratios indicated that much stronger selection occurred in hexaploid than in tetraploid wheats. Of course, the bottleneck effect at hexaploidization of wheat cannot be ignored as formation of common wheat was a very rare event (Feldman and Levy, 2015). Evidence for selection on the two AGPase genes in breeding was strong. Firstly, the frequency of *TaAGP‐S1‐7A‐Hap‐I* was about 20% in landraces, but nearly 80% in modern cultivars in the MCC, and the frequency of *TaAGP‐L‐1B‐Hap‐I* was less than 50% in landraces, but 85% in recent modern cultivars. Secondly, the frequencies of cultivars carrying favoured haplotypes also steadily increased among varieties released after the 1940s (Figure 5).

These favoured haplotypes were also selected in five other wheat production regions (Figure S6). Compared with yield‐related genes *TaTEF* and *TaCWI* (Jiang *et al*., 2015; Zheng *et al*., 2014) *TaAGP* had higher favoured haplotype frequencies and selection intensities. All these data indicated that *TaAGP* were critical genes selected for larger grain size during wheat domestication and breeding.

### Additive effects of major genes influencing grain weight in wheat

The TKWs of accessions combining the two favoured haplotypes were significantly higher than those of accessions carrying the nonfavoured haplotypes in both the MCC and MC (Figure S5), suggesting that genetically additive effects favoured selection at *TaAGP‐S1* and *TaAGP‐L*. *TaSus1* and *TaSus2*, another pair of key genes for enzymes involved in the starch synthesis pathway, showed a similar trend (Hou *et al*., 2014; Jiang *et al*., 2011). The four favoured haplotype cohorts showed highest TKW and positive selection in Chinese, European and North American cultivars (Figure 6). All of these examples indicate additive effects of major genes influencing TKW. Pyramiding these favoured haplotypes at multifunctional loci should be beneficial for future yield improvement in wheat.

## Conclusion

*TaAGP‐S1* and *TaAGP‐L* are important genes for enzymes involved in starch synthesis in developing endosperm. Haplotypes at *TaAGP‐S1‐7A* and *TaAGP‐L‐1B* are associated with TKW, and mean TKWs of favoured haplotypes are significantly higher than those of nonfavoured ones in different populations. Favoured haplotypes underwent strong positive selection during wheat domestication and breeding due to their additive genetic effects on TKW.

## Experimental procedures

### Plant materials

Two wheat populations were used: 245 accessions from the Chinese wheat Mini Core Collection (MCC) (Table S5) and 348 modern cultivars (MC) (Table S6) from the Chinese Wheat Core Collection (Hao *et al*., 2011). The MCC and MC were planted at the CAAS Luoyang Experiment Station in Henan province in 2002, 2005 and 2006, and CAAS Shunyi Experiment Station in Beijing in 2010.

Wheat NILs derived from an F_5_ population from cross Youzimai/Zhou 18*3//Handan 6172 were used for estimating SN, GN, and TKW and transcript level differences between *TaAGP‐S1‐7A‐Hap‐1* and *‐Hap‐2*. Another set of NILs from an F_4_ population from cross Yangmai 158/Zhou 18*3//Handan 6172 were used for measuring the same yield components and transcript level differences between *TaAGP‐L‐1B‐Hap‐1* and *‐Hap‐3* in 2014. Two NILs derived from a BC_3_F_5_ population from Isengrain/5*Yanzhan 4110 and a BC_3_F_6_ population of Jianmai/6*Zhou 18 were used for detecting TKW differences between *TaAGP‐L‐1B‐Hap‐1* and *‐Hap‐2*, and *TaAGP‐L‐1B‐Hap‐1* and *‐Hap‐4*, respectively. The data for all NILs are listed in Table S2.

Three hundred and eighty‐four European, 429 North American, 53 International Maize and Wheat Improvement Center (CIMMYT), 82 Russian and 51 Australian cultivars were surveyed to investigate the global distribution of favoured haplotypes of the *TaAGP‐S1‐7A* and *TaAGP‐L‐1B* genes and frequency changes of the favoured haplotypes during the last 100 years. Release dates and origins of all materials are provided in Hou *et al*. (2014).

Thirty‐seven accessions of diploid wheat and 36 accessions of tetraploid wheat were sequenced for evolutionary studies of *TaAGP‐S1‐7A* and *TaAGP‐L‐1B* (Table S7).

### Haplotype analysis

Primers were designed by Premier 5.0 (http://www.premierbiosoft.com/) and synthesized by BGI Tech (Shenzhen, China). PCR was conducted in reaction volumes of 15 mL containing 50–100 ng DNA, 7.5 mL buffer, 2.4 mL of 2.5 mm deoxynucleotide triphosphates, 0.5 mL of 10 mm forward and reverse primers, and 0.15 mL of *LA Taq* polymerase (TaKaRa Biotechnology). PCR was carried out in a Veriti 96‐Well Thermal Cycler (Applied Biosystems) with the following steps: denaturing at 95 °C for 5 min, followed by 32 cycles of 95 °C for 30 s, annealing at 55–63°C for 1 min, 72 °C for extension (1 kb/min) and a final extension of 72 °C for 10 min. PCR products were purified by purification kit DP‐1502 (TIANGEN), then cloned by the pGEM‐T Easy Cloning Vector (TIANGEN), and transformed into *Escherichia coli* cells by the heat shock method. Plasmids were extracted with a DP‐1002 Kit (TIANGEN). DNA was sequenced by an ABI 3730XI DNA Analyser (Applied Biosystems). Sequence alignments and SNP identification were made using DNASTAR (http://www.dnastar.com/). The digestion sites of restriction endonucleases were detected by Primer Premier 5.0. Two restriction endonucleases used in the study were produced by New England Biolabs (Beijing).

### Transcript analysis

RNA was extracted from developing grains of two near‐isogenic wheat lines (described in Plant materials) at 5, 10, 15, 20, 25 days postanthesis (DPA) using an RNAprep pure plant kit DP432 (TIANGEN) and reverse transcribed with an M‐MLV reverse transcriptase kit (Invitrogen, Shanghai, China). Relative real‐time PCR was carried out by Rotor‐Gene Q (Qiagen). Primers are listed in Table S1, and data are provided in Table S3.

### Promoter‐driven *GUS* expression in transgenic rice

About 1.4‐kb sequences upstream of the ATG start codon of two haplotypes at *TaAGP‐L‐1B* were amplified by primers L‐1B‐PEF and L‐1B‐PER (Table S1), and inserted into the pCAMBIA1391Z vector via *Sal*I and *EcoR*I digestion. The construct was mobilized into *Agrobacterium tumefaciens* EHA105 and then transformed into rice (*Oryza sativa* L. ssp. *japonica*) cv. Kitaake as described by Hiei *et al*. (1994). Wild‐type and transformed rice seedlings were planted at the CAAS Langfang Experiment Station in Hebei province in 2016. Each haplotype was represented by three positive transgenic lines, and 10 plants of each line were tested for *GUS* activity. *GUS* activities in developing seeds at 21 DPA were measured following the protocol of Hänsch *et al*. (1995). For each sample, 200 mg of fresh seeds was ground into powder in liquid nitrogen, and then, 1 mL of extraction solution (0.02885 m Na_2_HPO_4_, 0.02115 m NaH_2_PO_4_, 0.01 m EDTA, 0.1% SDS, 0.1% β‐mercaptoethanol, and 0.1% Triton × 100) was added, and centrifuged at 12 000 ***g*** for 10 min at 4 °C. Protein concentration in the supernatant was measured following the Bradford (1976) method. The supernatant was assayed for *GUS* activity with 4‐MUG (4‐methylumbelliferyl‐β‐D‐glucuronide) substrate as described by Hänsch *et al*. (1995). Fluorescence intensity levels were measured on a Tristar LB941 fluorescence spectrophotometer (Berthold) with 4‐MU (4‐methylumbelliferon) as the calibration control. GUS staining was performed following the protocol of Kosugi *et al*. (1990). The images of stained samples were captured using a Discovery V20 stereomicroscope (Carl Zeiss, Germany). All data are listed in Table S1.

### Statistical analysis

Data processing was carried out using Excel 2010 and SPSS Statistics 17.0 (http://www-01.ibm.com/software/analytics/spss/).

### Evolutionary studies

Diversity analysis and Tajima *D* tests of the two genes were carried out by DnaSP 5.10 (http://www.ub.es/dnasp). *F*_ST_ tests at *AGP‐S1‐7A* were carried out by Arlequin 3.5.1.2 (http://cmpg.unibe.ch/software/arlequin3).

## Conflict of interest

The authors declare that they have no conflict of interests.

## Supporting information


**Figure S1** Alignments of *TaAGP‐S1‐7A*,* ‐7B*,* ‐7D*,* TaAGP‐S2‐5A*,* ‐5B*,* ‐5D*,* TaAGP‐L‐1A*,* ‐1B* and *‐1D* based on cDNA (a) and genomic DNA (b) sequences.
**Figure S2** Mean TKWs of two genes in the MCC.
**Figure S3** Differences between *TaAGP‐S1‐7A‐Hap‐1* and *‐Hap‐2* in F_5_ NILs derived from Youzimai/Zhou 18*3//Handan 6172.
**Figure S4** Differences between *TaAGP‐L‐1B* haplotypes.
**Figure S5** Haplotype combinations in two populations.
**Figure S6** Global distribution of haplotypes.
**Figure S7** Dendrogram based on *AGP‐S1‐7A* sequences among diploid, tetraploid and hexaploid wheat accessions. Major polymorphic sites in tetraploid accessions are shown in the table below.

**Table S1** Primers used in this study.
**Table S2** Near‐isogenic lines derived from four combinations.
**Table S3** Haplotype transcript measurements at five stages of developing seeds.
**Table S4** *GUS* activity measurements in transgenic and wild type rice.
**Table S5** Accessions of hexaploid wheats in the Mini Core Collection.
**Table S6** Accessions of modern cultivars.
**Table S7** Progenitor accessions used in this study.

## References

[pbi12735-bib-0001] Arnold, K. , Bordoli, L. , Kopp, J. and Schwede, T. (2006) The SWISS‐MODEL workspace: a web‐based environment for protein structure homology modelling. Bioinformatics, 22, 195–201. 16301204 10.1093/bioinformatics/bti770

[pbi12735-bib-0002] Bahaji, A. , Li, J. , Sánchez‐López, Á.M. , Baroja‐Fernández, E. , Muñoz, F.J. , Ovecka, M. , Almagro, G. *et al*. (2014) Starch biosynthesis, its regulation and biotechnological approaches to improve crop yields. Biotechnol. Adv. 32, 87–106. 23827783 10.1016/j.biotechadv.2013.06.006

[pbi12735-bib-0003] Boehlein, S.K. , Shaw, J.R. , McCarty, D.R. , Hwang, S.K. , Stewart, J.D. and Hannah, L.C. (2013) The potato tuber, maize endosperm and a chimeric maize‐potato ADP‐glucose pyrophosphorylase exhibit fundamental differences in Pi inhibition. Arch. Biochem. Biophys. 537, 210–216. 23906662 10.1016/j.abb.2013.07.019

[pbi12735-bib-0004] Bradford, M.M. (1976) A rapid and sensitive method for the quantitation of microgram quantities of protein utilizing the principle of protein‐dye binding. Anal. Biochem. 72, 248–254. 942051 10.1016/0003-2697(76)90527-3

[pbi12735-bib-0005] Burton, R.A. , Johnson, P.E. , Beckles, D.M. , Fincher, G.B. , Jenner, H.L. , Naldrett, M.J. and Denyer, K. (2002) Characterization of the genes encoding the cytosolic and plastidial forms of ADP‐glucose pyrophosphorylase in wheat endosperm. Plant Physiol. 130, 1464–1475. 12428011 10.1104/pp.010363 PMC166665

[pbi12735-bib-0006] Cakir, B. , Tuncel, A. , Green, A.R. , Koper, K. , Hwang, S.K. , Okita, T.W. and Kang, C. (2015) Substrate binding properties of potato tuber ADP‐glucose pyrophosphorylase as determined by isothermal titration calorimetry. FEBS Lett. 589, 1444–1449. 25953126 10.1016/j.febslet.2015.04.042

[pbi12735-bib-0007] Comparot‐Moss, S. and Denyer, K. (2009) The evolution of the starch biosynthetic pathway in cereals and other grasses. J. Exp. Bot. 60, 2481–2492. 19505928 10.1093/jxb/erp141

[pbi12735-bib-0008] Copeland, L. and Preiss, J. (1981) Purification of spinach leaf ADPglucose pyrophosphorylase. Plant Physiol. 68, 996–1001. 16662079 10.1104/pp.68.5.996 PMC426033

[pbi12735-bib-0009] Corbi, J. , Debieu, M. , Rousselet, A. , Montalent, P. , Le Guilloux, M. , Manicacci, D. and Tenaillon, M.I. (2011) Contrasted patterns of selection since maize domestication on duplicated genes encoding a starch pathway enzyme. Theor. Appl. Genet. 122, 705–722. 21060986 10.1007/s00122-010-1480-9

[pbi12735-bib-0010] Denyer, K. , Dunlap, F. , Thorbjornsen, T. , Keeling, P. and Smith, A.M. (1996) The major form of ADP‐glucose pyrophosphorylase in maize endosperm is extra‐plastidial. Plant Physiol. 112, 779–785. 8883389 10.1104/pp.112.2.779 PMC158002

[pbi12735-bib-0011] Dickinson, D.B. and Preiss, J. (1969) ADP‐glucose pyrophosphorylase in shrunken‐2 and brittle‐2 mutants of maize endosperm. Plant Physiol. 44, 1058–1062. 16657157 10.1104/pp.44.7.1058 PMC396214

[pbi12735-bib-0012] Feldman, M. and Levy, A.A. (2015) Origin and evolution of wheat and related *Triticeae* species. In Alien Introgression in Wheat ( Molnár‐Láng, M. , Ceoloni, C. and Doležel, J. , eds), pp. 21–30. Switzerland: Springer International Publishing.

[pbi12735-bib-0013] Figueroa, C.M. , Kuhn, M.L. , Falaschetti, C.A. , Solamen, L. , Olsen, K.W. , Ballicora, M.A. and Iglesias, A.A. (2013) Unraveling the activation mechanism of the potato tuber ADP‐glucose pyrophosphorylase. PLoS ONE, 8, e66824. 23826149 10.1371/journal.pone.0066824 PMC3691274

[pbi12735-bib-0014] Georgelis, N. , Braun, E.L. , Shaw, J.R. and Hannah, L.C. (2007) The two AGPase subunits evolve at different rates in angiosperms, yet they are equally sensitive to activity‐altering amino acid changes when expressed in bacteria. Plant Cell, 19, 1458–1472. 17496118 10.1105/tpc.106.049676 PMC1913735

[pbi12735-bib-0015] Guex, N. and Peitsch, M.C. (1997) SWISS‐MODEL and the Swiss‐PdbViewer: an environment for comparative protein modeling. Electrophoresis, 18, 2714–2723. 9504803 10.1002/elps.1150181505

[pbi12735-bib-0016] Hannah, L.C. and Nelson, O.E. Jr . (1976) Characterization of ADP‐glucose pyrophosphorylase from shrunken‐2 and brittle‐2 mutants of maize. Biochem. Genet. 14, 547–560. 985379 10.1007/BF00485834

[pbi12735-bib-0017] Hänsch, R. , Koprek, T. , Mendel, R.R. and Schulze, J. (1995) An improved protocol for eliminating endogenous β‐glucuronidase background in barley. Plant Sci. 105, 63–69.

[pbi12735-bib-0018] Hao, C.Y. , Wang, L.F. , Ge, H.M. , Dong, Y.C. and Zhang, X.Y. (2011) Genetic diversity and linkage disequilibrium in Chinese bread wheat (*Triticum aestivum* L.) revealed by SSR markers. PLoS ONE, 6, e17279. 21365016 10.1371/journal.pone.0017279 PMC3041829

[pbi12735-bib-0019] Heldt, H.W. , Chon, C.J. and Maronde, D. (1977) Role of orthophosphate and other factors in the regulation of starch formation in leaves and isolated chloroplasts. Plant Physiol. 59, 1146–1155. 16660011 10.1104/pp.59.6.1146 PMC542524

[pbi12735-bib-0020] Hiei, Y. , Ohta, S. , Komari, T. and Kumashiro, T. (1994) Efficient transformation of rice (*Oryza sativa* L.) mediated by *Agrobacterium* and sequence analysis of the boundaries of the T‐DNA. Plant J. 6, 271–282. 7920717 10.1046/j.1365-313x.1994.6020271.x

[pbi12735-bib-0021] Hou, J. , Jiang, Q.Y. , Hao, C.Y. , Wang, Y.Q. , Zhang, H.N. and Zhang, X.Y. (2014) Global selection on sucrose synthase haplotypes during a century of wheat breeding. Plant Physiol. 164, 1918–1929. 24402050 10.1104/pp.113.232454 PMC3982753

[pbi12735-bib-0022] Hwang, S.K. , Nagai, Y. , Kim, D. and Okita, T.W. (2008) Direct appraisal of the potato tuber ADP‐glucose pyrophosphorylase large subunit in enzyme function by study of a novel mutant form. J. Biol. Chem. 283, 6640–6647. 18199755 10.1074/jbc.M707447200

[pbi12735-bib-0023] Jiang, Q.Y. , Hou, J. , Hao, C.Y. , Wang, L.F. , Ge, H.M. , Dong, Y.C. and Zhang, X.Y. (2011) The wheat (*T. aestivum*) sucrose synthase 2 gene (*TaSus2*) active in endosperm development is associated with yield traits. Funct. Integr. Genomics, 11, 49–61. 20821031 10.1007/s10142-010-0188-x

[pbi12735-bib-0024] Jiang, Y.M. , Jiang, Q.Y. , Hao, C.Y. , Hou, J. , Wang, L.F. , Zhang, H.N. , Zhang, S.N. *et al*. (2015) A yield‐associated gene *TaCWI*, in wheat: its function, selection and evolution in global breeding revealed by haplotype analysis. Theor. Appl. Genet. 128, 131–143. 25367379 10.1007/s00122-014-2417-5

[pbi12735-bib-0025] Jin, X. , Ballicora, M.A. , Preiss, J. and Geiger, J.H. (2005) Crystal structure of potato tuber ADP‐glucose pyrophosphorylase. EMBO J. 24, 694–704. 15692569 10.1038/sj.emboj.7600551 PMC549618

[pbi12735-bib-0026] Kang, G.Z. , Liu, G.Q. , Peng, X.Q. , Wei, L.T. , Wang, C.Y. , Zhu, Y.J. , Ma, Y. *et al*. (2013) Increasing the starch content and grain weight of common wheat by overexpression of the cytosolic AGPase large subunit gene. Plant Physiol. Biochem. 73, 93–98. 24080395 10.1016/j.plaphy.2013.09.003

[pbi12735-bib-0027] Kavakli, I.H. , Greene, T.W. , Salamone, P.R. , Choi, S.B. and Okita, T.W. (2001) Investigation of subunit function in ADP‐glucose pyrophosphorylase. Biochem. Biophys. Res. Commun. 281, 783–787. 11237727 10.1006/bbrc.2001.4416

[pbi12735-bib-0028] Kosugi, S. , Arai, Y. , Nakajima, K. and Ohashi, Y. (1990) An improved assay for β‐glucuronidase (GUS) in transformed cells: methanol almost suppresses a putative endogenous *GUS* activity. Plant Sci. 70, 133–140.

[pbi12735-bib-0029] Li, N. , Zhang, S.J. , Zhao, Y.J. , Li, B. and Zhang, J.R. (2011) Over‐expression of AGPase genes enhances seed weight and starch content in transgenic maize. Planta, 233, 241–250. 20978801 10.1007/s00425-010-1296-5

[pbi12735-bib-0030] Mariconti, L. , Pellegrini, B. , Cantoni, R. , Stevens, R. , Bergounioux, C. , Cella, R. and Albani, D. (2002) The E2F family of transcription factors from *Arabidopsis thaliana*. Novel and conserved components of the retinoblastoma/E2F pathway in plants. J. Biol. Chem. 277, 9911–9919. 11786543 10.1074/jbc.M110616200

[pbi12735-bib-0031] Müller‐Röber, B. , Sonnewald, U. and Willmitzer, L. (1992) Inhibition of the ADP‐glucose pyrophosphorylase in transgenic potatoes leads to sugar‐storing tubers and influences tuber formation and expression of tuber storage protein genes. EMBO J. 11, 1229–1238. 1373373 10.1002/j.1460-2075.1992.tb05167.x PMC556571

[pbi12735-bib-0032] Nagai, Y.S. , Sakulsingharoj, C. , Edwards, G.E. , Satoh, H. , Greene, T.W. , Blakeslee, B. and Okita, T.W. (2009) Control of starch synthesis in cereals: metabolite analysis of transgenic rice expressing an up‐regulated cytoplasmic ADP‐glucose pyrophosphorylase in developing seeds. Plant Cell Physiol. 50, 635–643. 19208694 10.1093/pcp/pcp021

[pbi12735-bib-0033] Neuhaus, H.E. , Kruckeberg, A.L. , Feil, R. and Stitt, M. (1989) Reduced‐activity mutants of phosphoglucose isomerase in the cytosol and chloroplast of Clarkia xantiana: II. Study of the mechanisms which regulate photosynthate partitioning. Planta, 178, 110–122. 24212556 10.1007/BF00392534

[pbi12735-bib-0034] Okita, T.W. , Nakata, P.A. , Anderson, J.M. , Sowokinos, J. , Morell, M. and Preiss, J. (1990) The subunit structure of potato tuber ADPglucose pyrophosphorylase. Plant Physiol. 93, 785–790. 16667537 10.1104/pp.93.2.785 PMC1062584

[pbi12735-bib-0035] Rose, M. , Huang, X.Q. and Brûlé‐Babel, A. (2015) Molecular characterization and sequence diversity of genes encoding the large subunit of the ADP‐glucose pyrophosphorylase in wheat (*Triticum aestivum* L.). J. Appl. Genet. 57, 15–25. 26109252 10.1007/s13353-015-0298-1

[pbi12735-bib-0036] Rossi, M. , Bermudez, L. and Carrari, F. (2015) Crop yield: challenges from a metabolic perspective. Curr. Opin. Plant Biol. 25, 79–89. 26002068 10.1016/j.pbi.2015.05.004

[pbi12735-bib-0037] Sakulsingharoj, C. , Choi, S. , Hwang, S. , Edwards, G. , Bork, J. , Meyer, C. , Preiss, J. *et al*. (2004) Engineering starch biosynthesis for increasing rice seed weight: the role of the cytoplasmic ADP‐glucose pyrophosphorylase. Plant Sci. 167, 1323–1333.

[pbi12735-bib-0038] Sarma, K. , Sen, P. , Barooah, M. , Choudhury, M.D. , Roychoudhury, S. and Modi, M.K. (2014) Structural comparison, substrate specificity, and inhibitor binding of AGPase small subunit from monocot and dicot: present insight and future potential. Biomed. Res. Int. 2014, 583606. 25276800 10.1155/2014/583606 PMC4167649

[pbi12735-bib-0039] Schwede, T. , Kopp, J. , Guex, N. and Peitsch, M.C. (2003) SWISS‐MODEL: an automated protein homology‐modeling server. Nucleic Acids Res. 31, 3381–3385. 12824332 10.1093/nar/gkg520 PMC168927

[pbi12735-bib-0040] Serrago, R.A. , Alzueta, I. , Savin, R. and Slafer, G.A. (2013) Understanding grain yield responses to source‐sink ratios during grain filling in wheat and barley under contrasting environments. Field. Crop. Res. 150, 42–51.

[pbi12735-bib-0041] Shi, J. and Lai, J. (2015) Patterns of genomic changes with crop domestication and breeding. Curr. Opin. Plant Biol. 24, 47–53. 25656221 10.1016/j.pbi.2015.01.008

[pbi12735-bib-0042] Smidansky, E.D. , Clancy, M. , Meyer, F.D. , Lanning, S.P. , Blake, N.K. , Talbert, L.E. and Giroux, M.J. (2002) Enhanced ADP glucose pyrophosphorylase activity in wheat endosperm increases seed yield. Proc. Natl Acad. Sci. USA, 99, 1724–1729. 11830676 10.1073/pnas.022635299 PMC122258

[pbi12735-bib-0043] Smidansky, E.D. , Martin, J.M. , Hannah, L.C. , Fischer, A.M. and Giroux, M.J. (2003) Seed yield and plant biomass increases in rice are conferred by deregulation of endosperm ADP‐glucose pyrophosphorylase. Planta, 216, 656–664. 12569408 10.1007/s00425-002-0897-z

[pbi12735-bib-0044] Stark, D.M. , Timmerman, K.P. , Barry, G.F. , Preiss, J. and Kishore, G.M. (1992) Regulation of the amount of starch in plant tissues by ADP glucose pyrophosphorylase. Science, 258, 287–292. 17835129 10.1126/science.258.5080.287

[pbi12735-bib-0045] Thorbjørnsen, T. , Villand, P. , Denyer, K. , Olsen, O.A. and Smith, A.M. (1996) Distinct isoforms of ADP‐glucose pyrophosphorylase occur inside and outside the amyloplasts in barley endosperm. Plant J. 10, 243–250.

[pbi12735-bib-0046] Thorneycroft, D. , Hosein, F. , Thangavelu, M. , Clark, J. , Vizir, I. , Burrell, M.M. and Ainsworth, C. (2003) Characterization of a gene from chromosome 1B encoding the large subunit of ADPglucose pyrophosphorylase from wheat: evolutionary divergence and differential expression of *Agp2* genes between leaves and developing endosperm. Plant Biotechnol. J. 1, 259–270. 17163903 10.1046/j.1467-7652.2003.00025.x

[pbi12735-bib-0047] Tsai, C.Y. and Nelson, O.E. (1966) Starch‐deficient maize mutant lacking adenosine diphosphate glucose pyrophosphorylase activity. Science, 151, 341–343. 5903344 10.1126/science.151.3708.341

[pbi12735-bib-0048] Vandepoele, K. , Raes, J. , De Veylder, L. , Rouze, P. , Rombauts, S. and Inzé, D. (2002) Genome‐wide analysis of core cell cycle genes in *Arabidopsis* . Plant Cell, 14, 903–916. 11971144 10.1105/tpc.010445 PMC150691

[pbi12735-bib-0049] Vandepoele, K. , Vlieghe, K. , Florquin, K. , Hennig, L. , Beemster, G.T. , Gruissem, W. , Van de Peer, Y. *et al*. (2005) Genome‐wide identification of potential plant E2F target genes. Plant Physiol. 139, 316–328. 16126853 10.1104/pp.105.066290 PMC1203381

[pbi12735-bib-0050] Wang, Z.Y. , Chen, X.P. , Wang, J.H. , Liu, T.S. , Liu, Y. , Zhao, L. and Wang, G.Y. (2007) Increasing maize seed weight by enhancing the cytoplasmic ADP‐glucose pyrophosphorylase activity in transgenic plants. Plant Cell Tissue Organ Cult. 88, 83–92.

[pbi12735-bib-0051] Zheng, J. , Liu, H. , Wang, Y.Q. , Wang, L.F. , Chang, X.P. , Jing, R.L. , Hao, C.Y. *et al*. (2014) *TEF‐7A*, a transcript elongation factor gene, influences yield‐related traits in bread wheat (*Triticum aestivum* L.). J. Exp. Bot. 65, 5351–5365. 25056774 10.1093/jxb/eru306 PMC4157721

