# Supplementary material for: ADP‐glucose pyrophosphorylase genes, associated with kernel weight, underwent selection during wheat domestication and breeding
Source: Plant Biotechnol J. 2017 May 12;15(12):1533–43. doi: 10.1111/pbi.12735 (PMC5698054; doi:10.1111/pbi.12735)
Supplement: Supplementary file 1 — Figure S1 Alignments of TaAGP‐S1‐7A, ‐7B, ‐7D, TaAGP‐S2‐5A, ‐5B, ‐5D, TaAGP‐L‐1A, ‐1B and ‐1D based on cDNA (a) and genomic DNA (b) sequences. Figure S2 Mean TKWs of two genes in the MCC. Figure S3 Differences between TaAGP‐S1‐7A‐Hap‐1 and ‐Hap‐2 in F5 NILs derived from Youzimai/Zhou 18*3//Handan 6172. Figure S4 Differences between TaAGP‐L‐1B haplotypes. Figure S5 Haplotype combinations in two populations. Figure S6 Global distribution of haplotypes. Figure S7 Dendrogram based on AGP‐S1‐7A sequences among diploid, tetraploid and hexaploid wheat accessions. Major polymorphic sites in tetraploid accessions are shown in the table below. [file PBI-15-1533-s002.pdf]

ADP-glucose pyrophosphorylase genes, associated with kernel weight, underwent selections during wheat domestication and breeding

Supplementary Figures

Figure legends

Figure S1 Alignments of *TaAGP-S1-7A, -7B, -7D, TaAGP-S2-5A, -5B, -5D, TaAGP-L-1A, -1B* and *-1D* based on cDNA (a) and genomic DNA (b) sequences.

Figure S2 Mean TKWs of two genes in the MCC. (a) TKWs of *TaAGP-S1-7A Hap-1, -2, -3* and *-4*. (b) TKWs of *TaAGP-L-1B Hap-1, -2, -3* and *-4*. (c) TKWs of *TaAGP-S1-7A-Hap-I* and *-Hap-II*. (d) TKWs of *TaAGP-L-1B-Hap-I* and *-Hap-II*.

* $P< 0.05$, ** $P< 0.01$, *** $P< 0.001$

Figure S3 Differences between *TaAGP-S1-7A-Hap-1* and *-Hap-2* in F_5 NILs derived from Youzimai/Zhou 18*3//Handan 6172. (a) SN, spike number per unit plant; GN, grain number per spike; TKW, thousand kernel weight in 2014 plot tests. (b) Relative expression levels in seeds at 5, 10, 15, 20 and 25 days post anthesis (DPA).

Figure S4 Differences between *TaAGP-L-1B* haplotypes. (a) SN, GN and TKW of *TaAGP-L-1B-Hap-1* and *-Hap-3* in F_4 NILs from Yangmai 158/Zhou 18*3//Handan 6172. (b) Relative expression levels of *TaAGP-L-1B-Hap-1* and *-Hap-3* in seeds at 5, 10, 15, 20 and 25 DPA. (c) TKWs of *TaAGP-L-1B-Hap-1* and *-Hap-2* in BC_3F_5 NILs from Isengrain/5*Yanzhan 4110. (d) TKWs of *TaAGP-L-1B-Hap-1* and *-Hap-4* in BC_3F_6 NILs from Jianmai/6*Zhou 18.

* $P< 0.001$

Figure S5 Haplotype combinations in two populations. (a) Mean TKWs of haplotype combinations among MCC accessions. (b) Mean TKWs of haplotype combinations among MC accessions. (c) Frequencies of haplotype combinations among MC accessions released over six decades.

SI, SII: *TaAGP-S1-7A-Hap-I*, *-Hap-II*. LI, LII: *TaAGP-L-1B-Hap-I*, *-Hap-II*.

* $P < 0.05$, ** $P < 0.01$, *** $P < 0.001$

Figure S6 Global distribution of haplotypes. (a) Frequencies of haplotypes at *TaAGP-S1-7A* in six major wheat production regions. (b) Frequencies of haplotypes at *TaAGP-L-1B* in the same six regions. (c) TKW difference between favored and non-favored haplotypes at six loci in the MC in 2002, 2006 and 2010. (d) Favored haplotype frequencies at six loci in Chinese cultivars (CC), European cultivars (EC) and North American cultivars (NAC).

Figure S7 Dendrogram based on *AGP-S1-7A* sequences among diploid, tetraploid and hexaploid wheat accessions. Major polymorphic sites in tetraploid accessions are shown in the table below.

AR, *Triticum araraticum*; BO, *T. boeoticum*; DM, *T. dicoccum*; DR, *T. durum*; DS, *T. dicoccoides*; MO, *T. monococcum*; PO, *T. polonicum*; PS, *T. persicum*; TG, *T. turgidum* ; TR, *T. orientale*; UR, *T. urartu*.

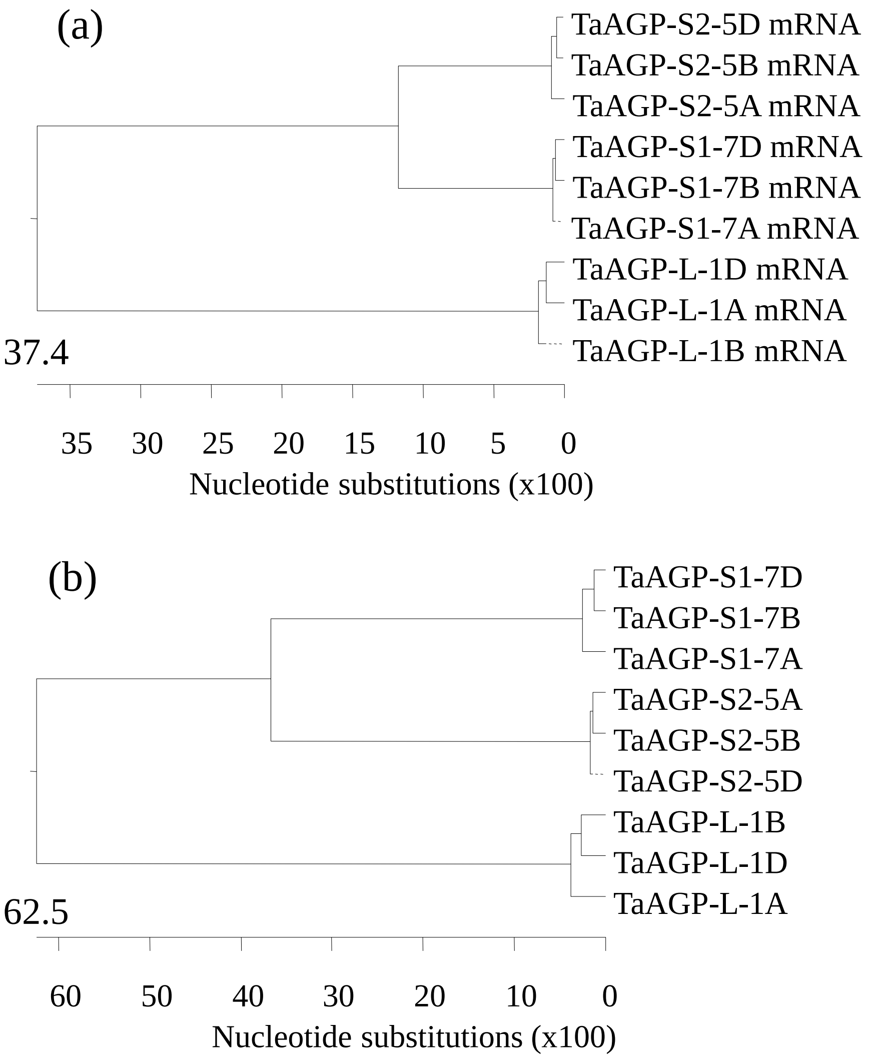
(a)
TaAGP-S2-5D mRNA
TaAGP-S2-5B mRNA
TaAGP-S2-5A mRNA
TaAGP-S1-7D mRNA
TaAGP-S1-7B mRNA
TaAGP-S1-7A mRNA
TaAGP-L-1D mRNA
TaAGP-L-1A mRNA
TaAGP-L-1B mRNA
37.4
35 30 25 20 15 10 5 0
Nucleotide substitutions (x100)
(b)
TaAGP-S1-7D
TaAGP-S1-7B
TaAGP-S1-7A
TaAGP-S2-5A
TaAGP-S2-5B
TaAGP-S2-5D
TaAGP-L-1B
TaAGP-L-1D
TaAGP-L-1A
62.5
60 50 40 30 20 10 0
Nucleotide substitutions (x100)

Figure S1

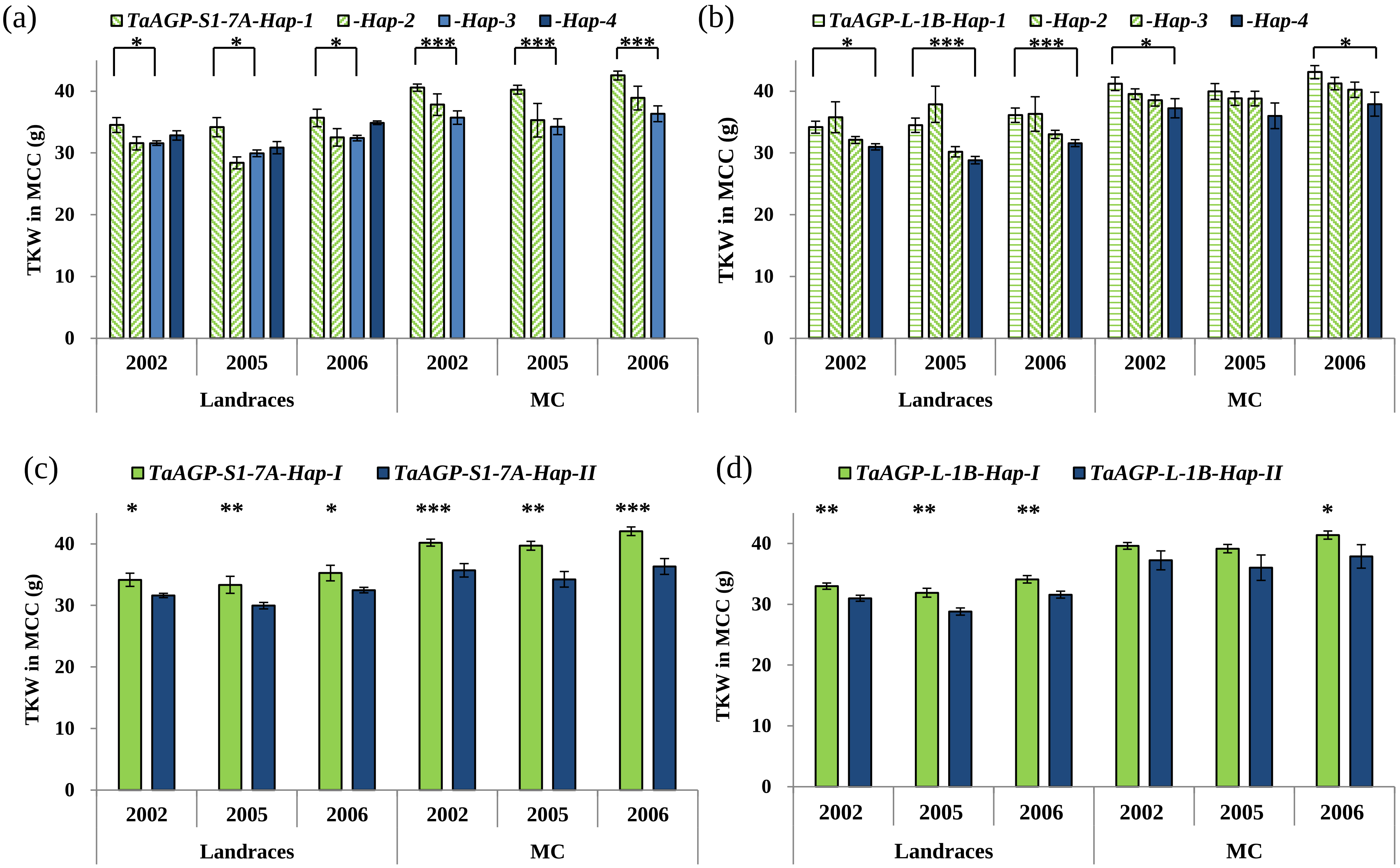
(a)
TaAGP-S1-7A-Hap-1
-Hap-2
-Hap-3
-Hap-4
*
*
*
***
***
***
TKW in MCC (g)
0
10
20
30
40
2002
2005
2006
2002
2005
2006
Landraces
MC
(b)
TaAGP-L-1B-Hap-1
-Hap-2
-Hap-3
-Hap-4
*
***
***
*
*
TKW in MCC (g)
2002
2005
2006
2002
2005
2006
Landraces
MC
(c)
TaAGP-S1-7A-Hap-I
TaAGP-S1-7A-Hap-II
*
**
*
***
**
***
TKW in MCC (g)
2002
2005
2006
2002
2005
2006
Landraces
MC
(d)
TaAGP-L-1B-Hap-I
TaAGP-L-1B-Hap-II
**
**
**
*
TKW in MCC (g)
2002
2005
2006
2002
2005
2006
Landraces
MC

Figure S2

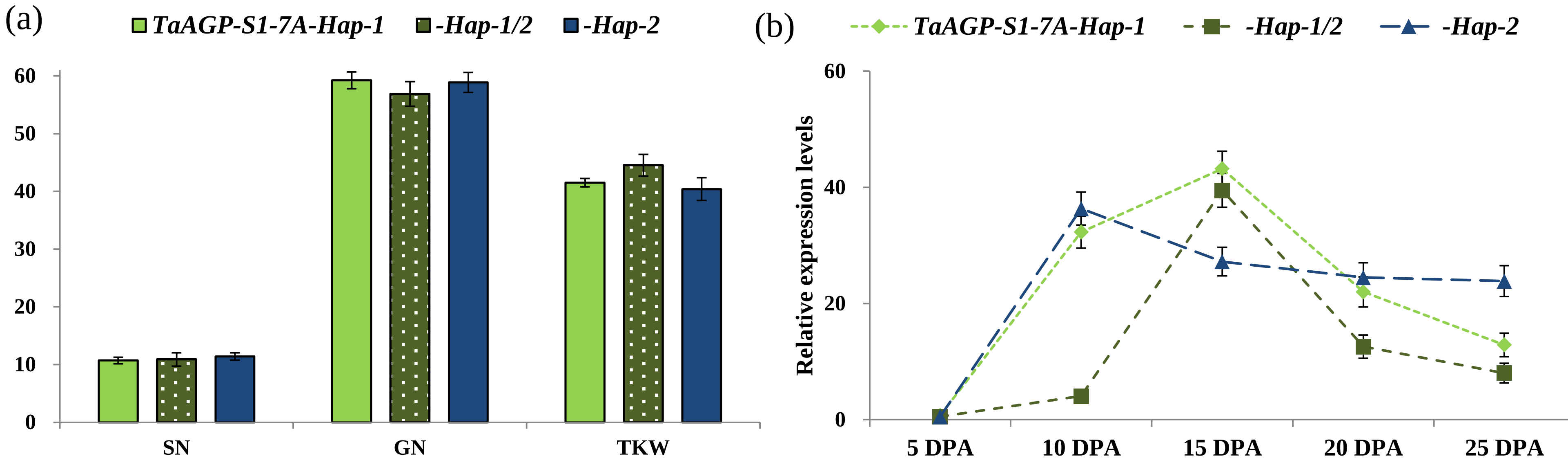

(a)
TaAGP-S1-7A-Hap-1
-Hap-1/2
-Hap-2
60
50
40
30
20
10
0
SN
GN
TKW
(b)
TaAGP-S1-7A-Hap-1
-Hap-1/2
-Hap-2
Relative expression levels
60
40
20
0
5 DPA
10 DPA
15 DPA
20 DPA
25 DPA

Figure S3

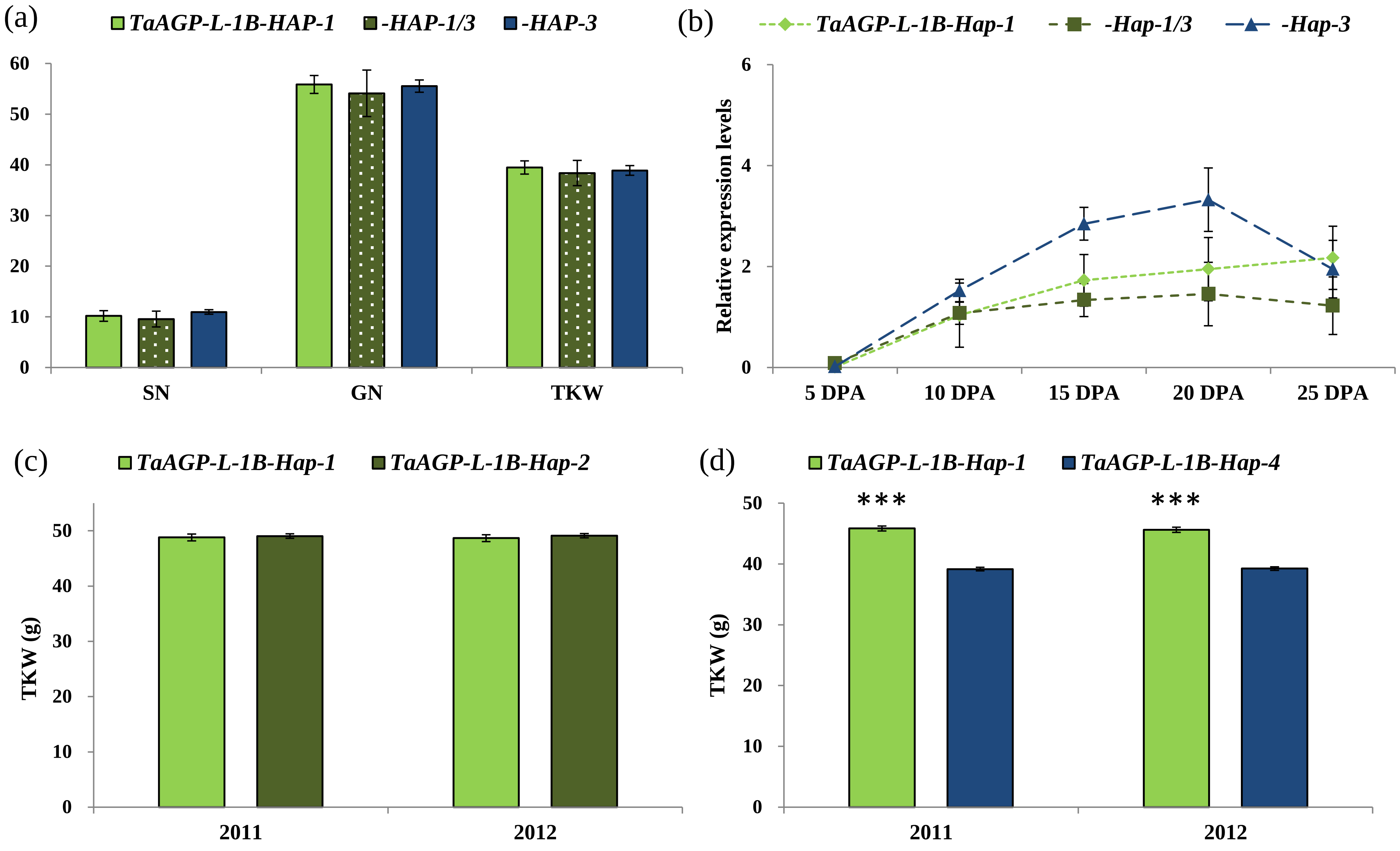
(a)
TaAGP-L-1B-HAP-1
-HAP-1/3
-HAP-3
60
50
40
30
20
10
0
SN
GN
TKW
(b)
TaAGP-L-1B-Hap-1
-Hap-1/3
-Hap-3
Relative expression levels
6
4
2
0
5 DPA
10 DPA
15 DPA
20 DPA
25 DPA
(c)
TaAGP-L-1B-Hap-1
TaAGP-L-1B-Hap-2
TKW (g)
50
40
30
20
10
0
2011
2012
(d)
TaAGP-L-1B-Hap-1
TaAGP-L-1B-Hap-4
TKW (g)
***
***
50
40
30
20
10
0
2011
2012

Figure S4

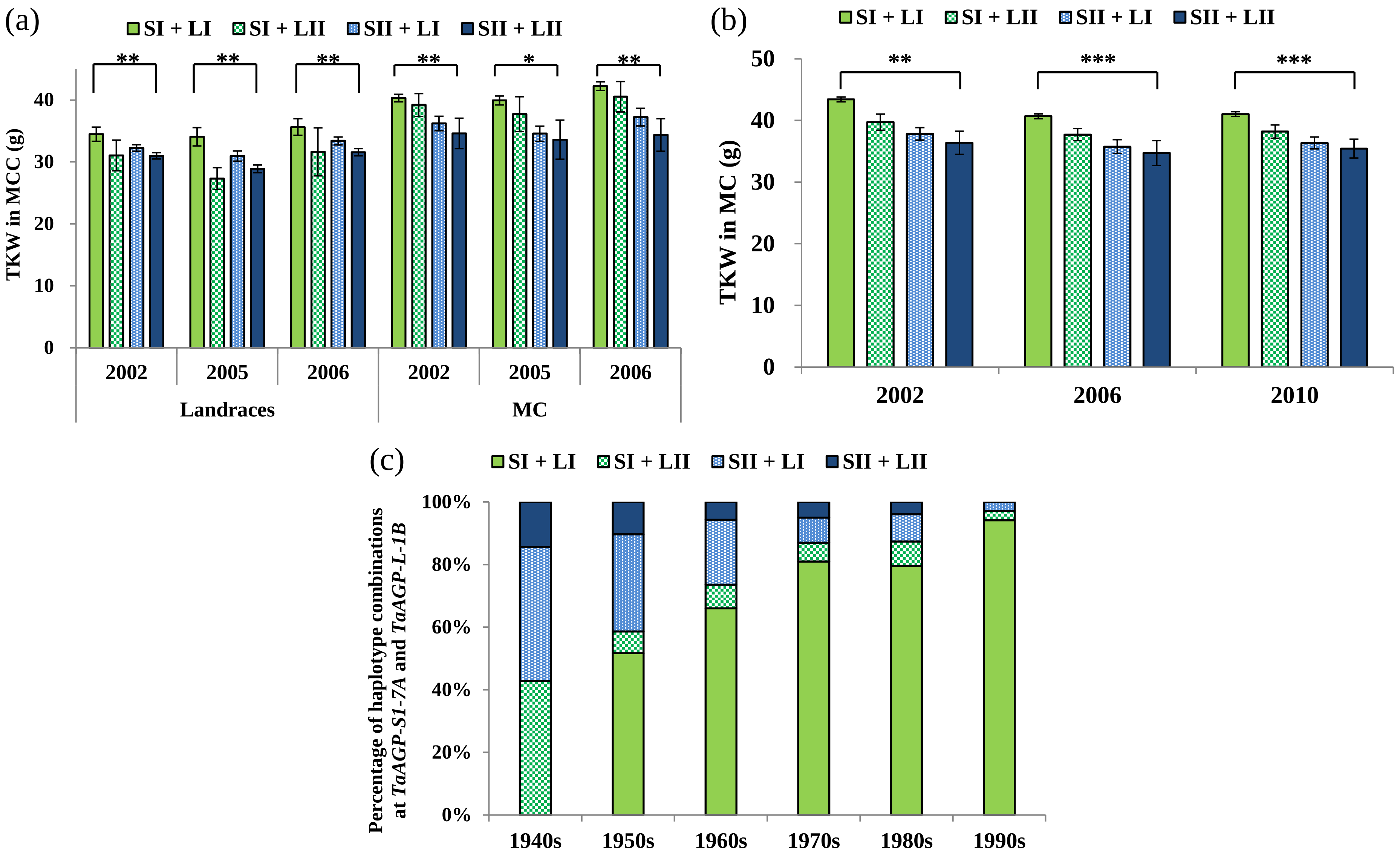
(a)
SI + LI
SI + LII
SII + LI
SII + LII
TKW in MCC (g)
** ** ** ** * **
2002
2005
2006
2002
2005
2006
Landraces
MC
(b)
TKW in MC (g)
** *** ***
2002
2006
2010
(c)
Percentage of haplotype combinations at *TaAGP-S1-7A* and *TaAGP-L-1B*
1940s
1950s
1960s
1970s
1980s
1990s

Figure S5

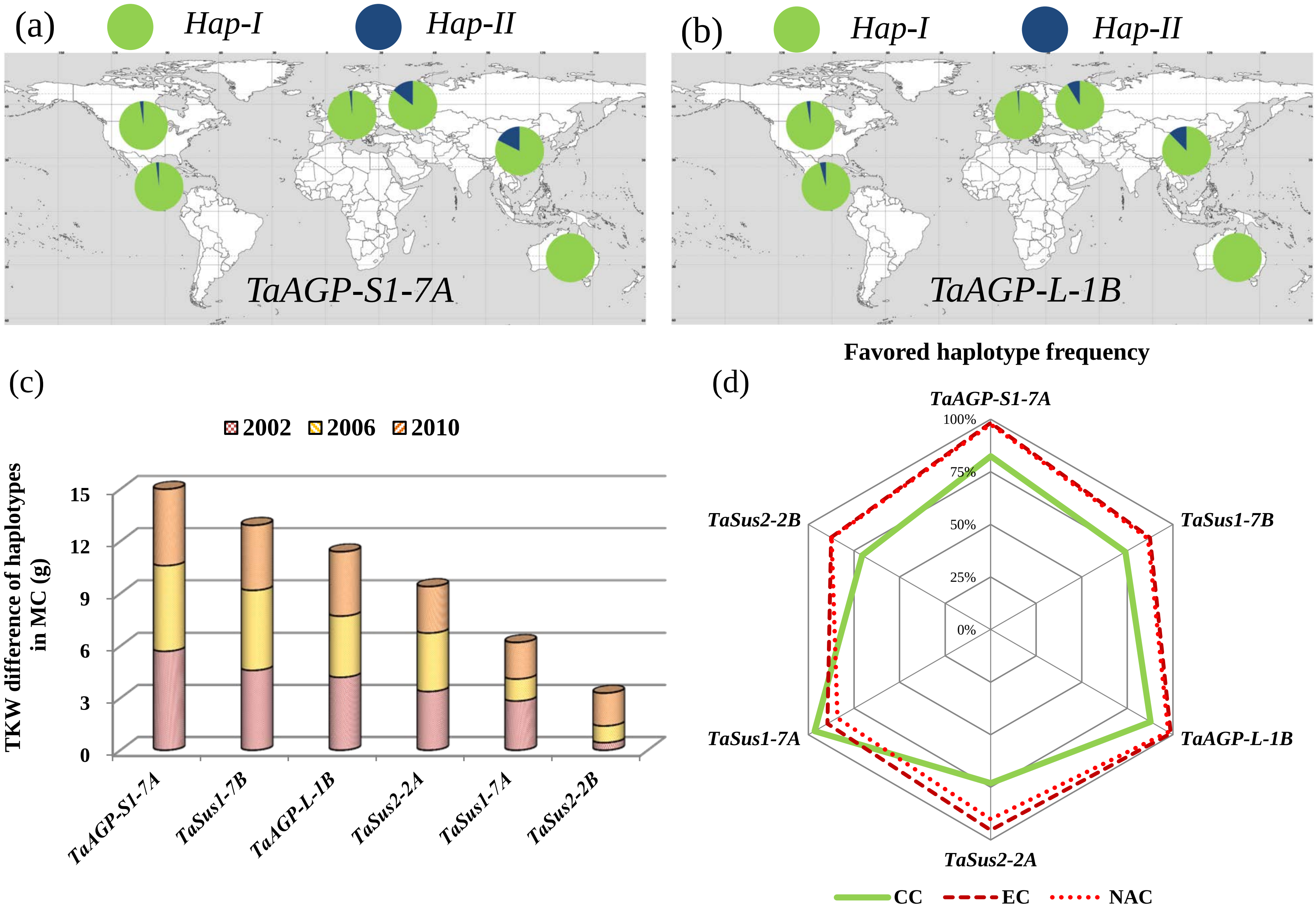

(a)
Hap-I
Hap-II
TaAGP-S1-7A
(b)
Hap-I
Hap-II
TaAGP-L-1B
(c)
2002
2006
2010
TKW difference of haplotypes in MC (g)
0
3
6
9
12
15
TaAGP-S1-7A
TaSus1-7B
TaAGP-L-1B
TaSus2-2A
TaSus1-7A
TaSus2-2B
Favored haplotype frequency
(d)
TaAGP-S1-7A
100%
75%
50%
25%
0%
TaSus1-7B
TaAGP-L-1B
TaSus2-2A
TaSus1-7A
TaSus2-2B
CC
EC
NAC

Figure S6

Figure S7

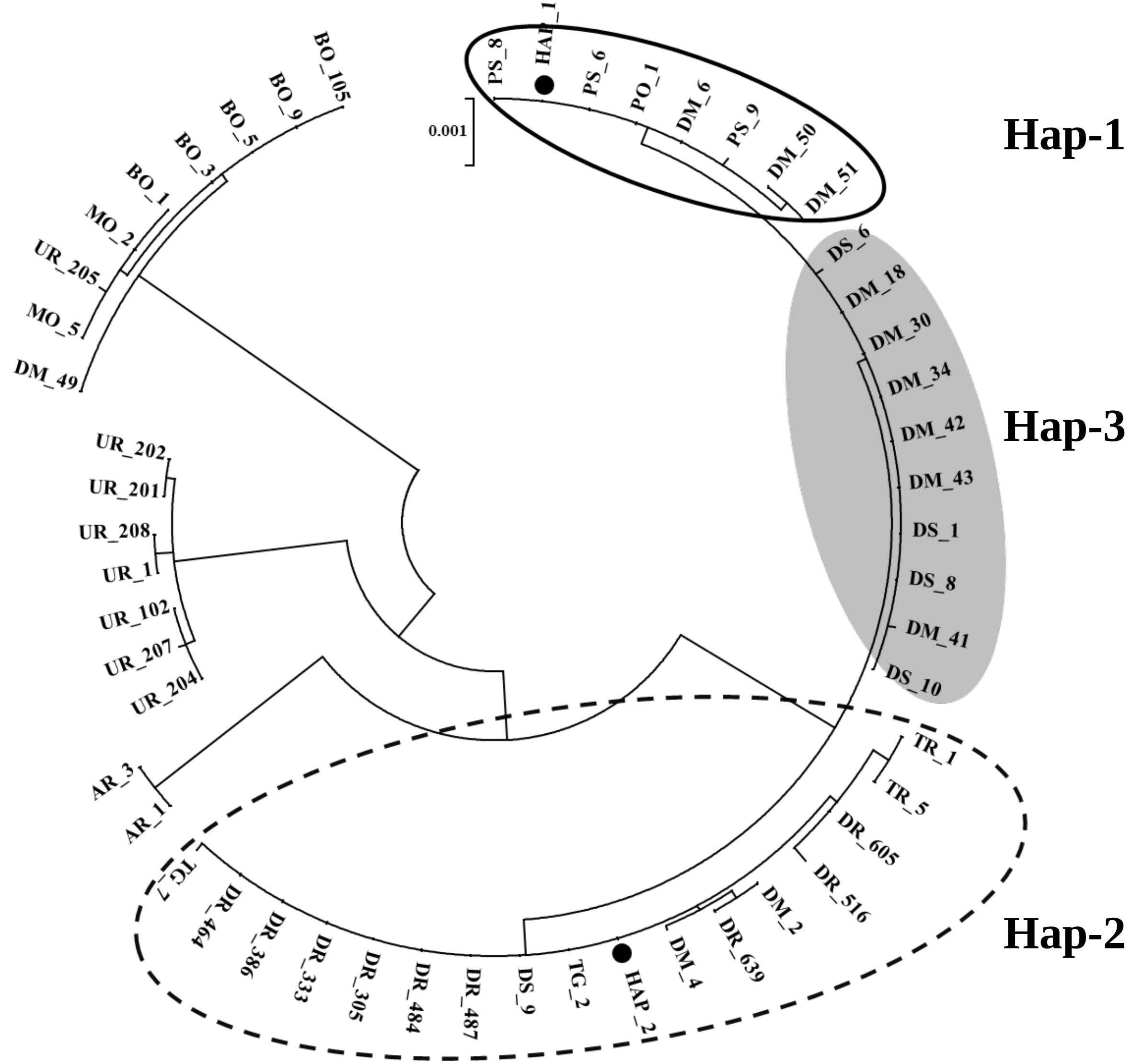

	929	943	1034	2267	2716	3191	3237	3404	3528	3597	7193
**Hap-1**	G	G	T	A	T	T	C	T	T	A	-
**Hap-2**	A	A	T	-	C	C	T	C	G	G	TCCTATC
**Hap-3**	G	G	G	A	T	C	C	C	G	A	-
